# Novel tumor necrosis factor-related long non-coding RNAs signature for risk stratification and prognosis in glioblastoma

**DOI:** 10.3389/fneur.2023.1054686

**Published:** 2023-04-20

**Authors:** Shengrong Long, Bingbing Wu, Liu Yang, Lesheng Wang, Bo Wang, Yu Yan, Jiazhi Jiang, Bin Yang, Qiangqiang Zhou, Min Shi, Wu Liang, Wei Wei, Xiang Li

**Affiliations:** ^1^Department of Neurosurgery, Zhongnan Hospital of Wuhan University, Wuhan, China; ^2^Brain Research Center, Zhongnan Hospital of Wuhan University, Wuhan, China; ^3^Department of Neurosurgery, Yongchuan Hospital of Chongqing Medical University, Chongqing, China; ^4^Department of Neurosurgery, Central Theater General Hospital of the Chinese People's Liberation Army, Wuhan, China; ^5^Department of Neurosurgery, The Affiliated Minda Hospital of Hubei University for Nationalities, Enshi, China; ^6^Medical Research Institute, Wuhan University, Wuhan, China

**Keywords:** TNF-related risk signature, glioblastoma multiforme, immune, biomarker, prognosis

## Abstract

**Background:**

Tumor necrosis factor (TNF) is an inflammatory cytokine that can coordinate tissue homeostasis by co-regulating the production of cytokines, cell survival, or death. It widely expresses in various tumor tissues and correlates with the malignant clinical features of patients. As an important inflammatory factor, the role of TNFα is involved in all steps of tumorigenesis and development, including cell transformation, survival, proliferation, invasion and metastasis. Recent research has showed that long non-coding RNAs (lncRNAs), defined as RNA transcripts >200 nucleotides that do not encode a protein, influence numerous cellular processes. However, little is known about the genomic profile of TNF pathway related-lncRNAs in GBM. This study investigated the molecular mechanism of TNF related-lncRNAs and their immune characteristics in glioblastoma multiforme (GBM) patients.

**Methods:**

To identify TNF associations in GBM patients, we performed bioinformatics analysis of public databases - The Cancer Genome Atlas (TCGA) and the Chinese Glioma Genome Atlas (CGGA). The ConsensusClusterPlus, CIBERSORT, Estimate, GSVA and TIDE and first-order bias correlation and so on approaches were conducted to comprehensively characterize and compare differences among TNF-related subtypes.

**Results:**

Based on the comprehensive analysis of TNF-related lncRNAs expression profiles, we constructed six TNF-related lncRNAs (C1RL-AS1, LINC00968, MIR155HG, CPB2-AS1, LINC00906, and WDR11-AS1) risk signature to determine the role of TNF-related lncRNAs in GBM. This signature could divide GBM patients into subtypes with distinct clinical and immune characteristics and prognoses. We identified three molecular subtypes (C1, C2, and C3), with C2 showing the best prognosis; otherwise, C3 showing the worst prognosis. Moreover, we assessed the prognostic value, immune infiltration, immune checkpoints, chemokines cytokines and enrichment analysis of this signature in GBM. The TNF-related lncRNA signature was tightly associated with the regulation of tumor immune therapy and could serve as an independent prognostic biomarker in GBM.

**Conclusion:**

This analysis provides a comprehensive understanding of the role of TNF-related characters, which may improve the clinical outcome of GBM patients.

## 1. Introduction

Gliomas account for approximately 80% of primary malignant tumors in the central nervous system, and the majority are the most malignant glioblastoma multiforme (GBMs) (1). Currently, the conventional clinical treatment protocol is maximal tumor resection with the preservation of functional areas and the standard adjuvant treatment method of temozolomide (TMZ) combined with radiotherapy after the operation (2). Even so, the prognosis for GBM patients is poor, especially for isocitrate dehydrogenase (IDH) wild-type, with a mean overall survival time of <14.6 months for newly diagnosed GBM patients and 6.9 months for recurrent GBM patients, the five-year survival rate of less than 10% (3).

Recently, many new treatment strategies for glioma have emerged, notably, the tumor treating fields (TTF) approved in China in 2020 (4). In addition, immune checkpoint blockade (ICB) drugs have drawn widespread attention. Immune checkpoint inhibitors against programmed cell death protein 1 (PD1) and programmed cell death 1 ligand 1 (PD-L1) belong to the B7-CD28 family (5) and are the best-described immunotherapy targets. However, most ICB drugs represented by anti-PD-1 have been tested in glioma clinical trials and did not achieve the desired effect in clinical trials, suggesting the presence of another costimulatory signal in the tumor microenvironment in GBM. Recently, it has been shown that in addition to blocking the co-suppressive immune checkpoints of the B7-CD28 family, promoting T cell responses by binding to the tumor necrosis factor (TNF) family of costimulatory receptors is another potential therapeutic approach (6). The TNF family consists of 19 TNF ligand superfamilies (TNFSFs) and 29 TNF receptor superfamilies (TNFRSFs) (7), which mediate the signal transduction that controls the survival, proliferation, differentiation and effector functions of immune cells and non-immune cells (8). TNF family members usually display proinflammatory functions through activation of the NF-kB pathway. Activating the TNFSF/TNFRSF family may also trigger cell apoptosis or other forms of cell death, leading to the activation or suppression of immune responses in the tumor microenvironment (9). Thus, modulating the interaction between the TNFSF/TNFRSF family has great potential for cancer therapy. Many therapeutic approaches targeting the TNF family, including CD40, OX40, 4-1BB, GITR, and CD27, are currently being actively investigated in clinical trials in various cancers, including GBM, lung cancer, malignant pleural mesothelioma, and spontaneous prostate cancer (7, 10–12). However, the expression patterns and clinical significance of TNF members related-lncRNAs in GBM have not yet been evaluated.

Long non-coding RNAs (lncRNAs) are those greater than 200 nucleotides (nt) in length without coding functions. More than 15,000 lncRNAs have been identified in the human genome, including intronic noncoding RNAs, large intergenic RNAs (lincRNAs), antisense RNAs and pseudogenes (13). lncRNAs can affect life activities in a variety of ways, such as affecting the transcriptional regulation of nearby genes, binding to proteins and regulating their functions, or acting as competing endogenous RNAs (ceRNAs), which are affected by adsorbing microRNA (miRNA) target gene translation. The action mode of lncRNAs is closely related to their subcellular location (14). The lncRNAs in the nucleus are mostly involved in transcriptional regulation or chromatin remodeling, while those in the cytoplasm are mostly involved in the posttranslational regulation of mRNAs or binding to corresponding proteins and affecting their activities. Recent studies have revealed that some lncRNA sequences contain short open reading frames (ORFs), which could encode short peptides and involve important life events (15). In recent years, the role of lncRNAs in tumorigenesis and development has been gaining attention in the industry, as the emergence of lncRNAs has greatly expanded the depth of research in tumor genomics. Recent studies have found that abnormal lncRNA expression has extensive effects on malignant characteristics of GBM, such as proliferation, apoptosis, invasion, and chemoradiotherapy resistance. Up-regulated tumor-promoting lncRNAs such as MALAT1, UCA1, H19, HIF1A-AS2 and down-regulated tumor suppressor lncRNAs such as NBAT1, GASS, RNCR3, etc., all of which play an important role in the formation and malignant progression of gliomas (16, 17). Clarifying the abnormal expression patterns and regulatory mechanism of lncRNAs provide a new theoretical basis for the treatment of glioma and brought hope to overcome glioma.

**Figure 1:**
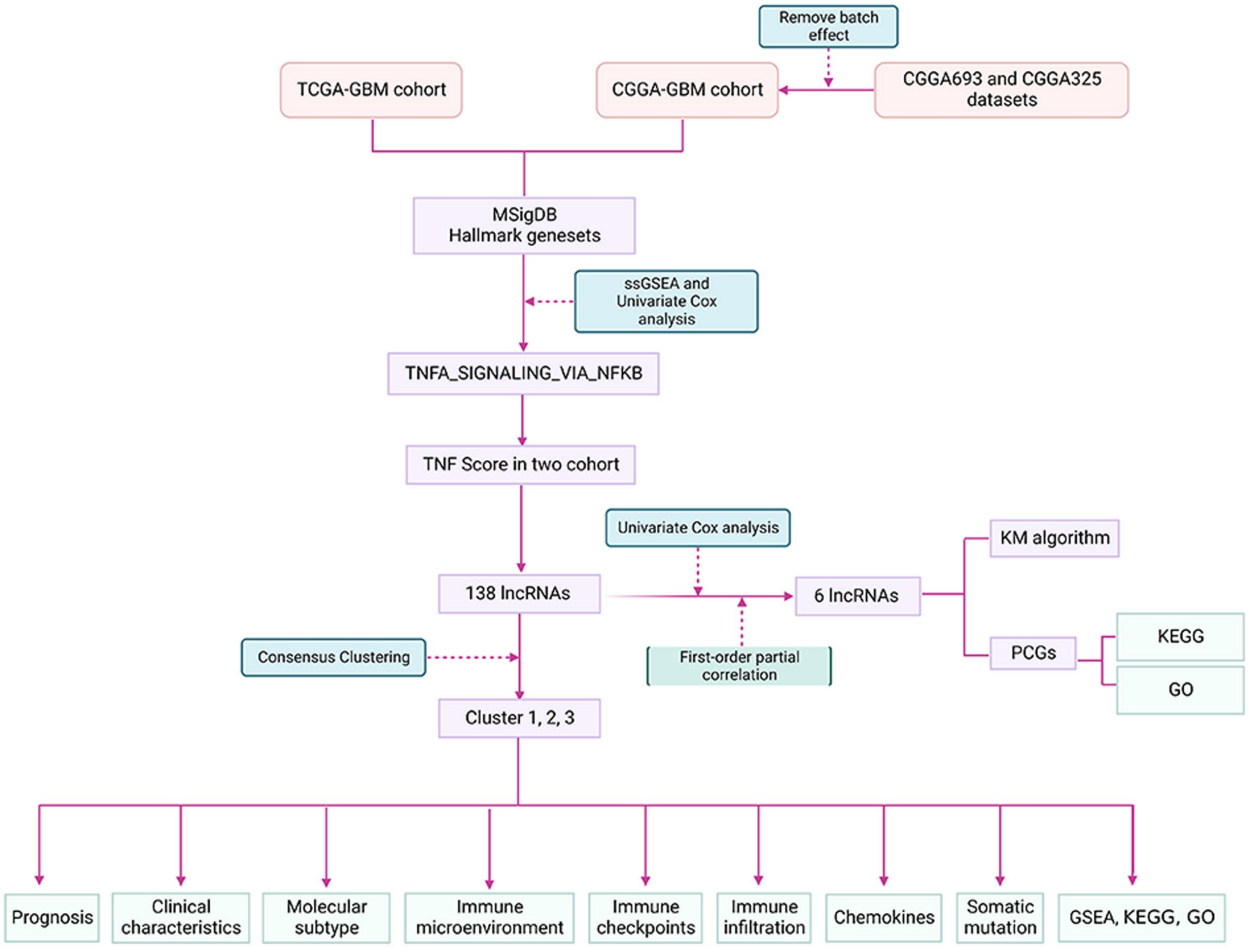
The flow chart of the study.

WHO Classification of Tumors of the Central Nervous System (WHO CNS5) (18) has been used to predict the prognosis of patients with glioma for many years. It is still sometimes inaccurate, given the heterogeneity of the tumors. In addition to identifying potential biomarkers, new advances in bioinformatics and genome sequencing technologies have also helped predict the prognosis of cancer patients (8, 19). Many studies have shown that individual lncRNA biomarkers have limited prognostic value and that integrating multiple biomarkers into a single model is better (16). For example, based on a network of metastasis-related ceRNAs, three lncRNAs can predict the prognosis of colorectal cancer (CRC) (20). By analyzing TCGA lung cancer datasets, four lncRNA signatures could effectively predict the survival time of lung adenocarcinoma (LUAD) (21). In this study, we developed and validated a prognostic signature based on TNF pathway-related lncRNAs using more than 1,000 samples from three cohorts. Considering the unique role of the TNF pathway in the interaction between tumor-infiltrating lymphocytes and tumor cells, as well as its potential role in controlling the body’s response to immunotherapy, we further explored the relationship between features and immune-related profiles, as well as their association with immune treatment response. Therefore, a better understanding of the TNF-related lncRNAs in GBM and the clinical and immunological characteristics based on its signature may help optimize cancer immunotherapy. Therefore, we hypothesized that the six TNF-related lncRNAs as a prognostic model might also contribute to the discovery of prognosis-related biomarkers in glioma patients. The findings may provide new insights to develop creative therapeutic strategies for GBM. The flow chart of our study is depicted in Figure 1.

## 2. Materials and methods

### 2.1. Datasets download

The RNA-Seq data and clinical information of GBM and non-matched healthy controls were downloaded from The Cancer Genome Atlas (TCGA) Genomic Data Commons (GDC)^1^. In addition, we obtained expression and clinical information data from the Chinese Glioma Genome Atlas (CGGA) database^2^ of the CGGA693 and CGGA325 datasets (22).

### 2.2. Sources of related genes

Our access to the Gene Set Enrichment Analysis (GSEA) website obtains the TNF pathway gene sets in the h.all.v7.4.symbols.gmt file.

### 2.3. Data preprocessing

#### 2.3.1. TCGA data preprocessing

The following steps to preprocess RNA-seq data from TCGA:

(1) Removal of samples without survival information; retention of tumor samples and normal samples;

(2) Matching the ENSG to the Gene Symbol.

#### 2.3.2. CGGA data preprocessing

The following preprocessing steps were performed on the CGGA693 and CGGA325 datasets:

(1) Download and normalize the datasets;

(2) Retaining samples with survival time and status;

(3) Merging and normalized these two datasets: CGGA693 and CGGA325;

(4) Use the removeBatchEffect function of the limma R package to remove the batch effect between the two datasets, hereafter referred to as CGGA (Supplementary Figure S1). For the statistical information after processing the three datasets Supplementary Table S1 was found.

### 2.4. Acquisition of lncRNA expression profiles

The Version 37 of the GTF file was obtained from the GENCODE website^3^ and classified the TCGA and CGGA expression profiles into mRNA and lncRNA according to the annotations in the file.

### 2.5. Identification of critical HALLMARK pathways in glioblastoma

HALLMARK pathway scores were calculated by Single sample GSEA (ssGSEA) for TCGA and CGGA samples separately. For the scoring of the TCGA dataset, the rank sum test was used to identify the difference in the pathway scores between normal and tumor samples (Supplementary Datasheet S1), to identify the HALLMARK pathways related to prognosis through univariate analysis, and to take their intersection (Supplementary Datasheet S2). For the scores in the CGGA dataset, the prognosis-related HALLMARK pathway was identified through univariate analysis (Supplementary Datasheet S3). Then, HALLMARK pathways that were important in different datasets were found TNFA_SIGNALING_VIA_NFKB.

### 2.6. Identification of TNF-related lncRNAs

The Pearson correlation coefficient and *p* value were calculated for TCGA and CGGA of the TNF scores of the GBM samples in the corresponding dataset with lncRNAs. Filtered according to the correlation |cor| > 0.25 and *p* < 0.05 in these datasets.

### 2.7. Identification of TNF-related lncRNA subtypes

Consensus Cluster Plus is used to construct a consistency matrix to classify the samples (23). TNF score-related lncRNAs were obtained to obtain the TNF score-related lncRNA subtypes of the sample. Here, we utilized the KM algorithm and Euclidean as a metric of distance and performed 500 bootstraps, with each bootstrapping process including 80% of the patients in the training set. The number of clusters was set from 2 to 10, and the best classification was determined by calculating the consistency matrix and the consistency cumulative distribution function.

### 2.8. Gene set enrichment analysis and functional annotation of each subtype

To determine the pathways of different molecular subtypes in biological processes, we used GSEA for pathway analysis. We used all candidate gene sets in the Kyoto Encyclopedia of Genes and Genomes (KEGG) database for GSEA (24). WebGestaltR (V0.4.4) and the R package were used for the functional annotation of differential genes.

### 2.9. Analysis of the tumor immune microenvironment

The tumor immune microenvironment scores of the samples were evaluated by ESTIMATE, MCP-counter and ssGSEA and then compared their differential distribution in different subtypes.

### 2.10. First-order partial correlation of TNF-related lncRNAs

A first-order partial correlation was performed to explore interlinks among these TNF-related lncRNAs, TNF scores, and TNF-related genes. The TNF score was assumed to be *x*, and TNF- related gene expression was *y*. The first-order partial correlation between x and y conditioned on TNF-lncRNAs was


$$rxylncRNA=\frac{\mathrm{rxy}-\mathrm{rxlncRNA}*\mathrm{rylncRNA}}{\sqrt{\left(1-r2\mathrm{xlncRNA}\right)*\left(1-r2\mathrm{ylncRNA}\right)}}$$

### 2.11. Enrichment analysis of the TNF-related lncRNAs

The correlation between the key lncRNAs we screened and protein-coding genes (PCGs) were calculated in TCGA and CGGA and then filtered with Corr >0.4 and *p* < 0.05 as the threshold to obtain the PCGs positively correlated with lncRNAs and then took their intersection while performing functional enrichment analysis using the timeWebGestaltR (V0.4.4) R package.

### 2.12. Construction and validation of the TNF-related lncRNAs signature

In view of whether the vital lncRNA has prognostic power, we conducted a multivariate analysis in the TCGA dataset and obtained correlation coefficients for each lncRNA using the following formula to calculate a risk score for each patient: Score = (beta_i_ × Exp_i_), i refers to the expression level of TNF-related lncRNAs, beta is corresponding lncRNA in multivariate cox regression of the coefficient for that gene. Patients were divided into high- and low-risk groups based on the median value. The Kaplan–Meier method was used to draw survival curves for prognostic analysis, and the log-rank test was used to determine the significance of the differences. To verify the prognostic roles of this TNF-related lncRNAs signature, the nomogram, decision curve analysis (DCA) curve, calibration analysis and time-dependent receiver operating characteristic (ROC) curve was drawn to evaluate the predictive value of the prognostic gene signature for overall survival.

### 2.13. Cell culture, RNA extraction, cDNA synthesis, and quantitative real-time PCR

The human glioma cell lines, U251, T98G, and human astroglia SVGp12 were purchased from the Procell (Wuhan, China) and were cultured in Dulbecco’s modified Eagle medium (DMEM; Servicebio) with 10% fetal bovine serum (FBS, Excel) and 10ul/ml Penicillin–Streptomycin Solution, 100× (Biosharp) at a cell incubator (Hair) at 37°C with 5% CO_2_.

Takara RNAiso Plus (Takara Bio. Inc., Otsu, Shiga, Japan) was used to isolate total RNA from the tissues persevered at a temperature < −80°C according to the manual protocol. cDNA was synthesized from 1ug RNA using the HiScriptIIQ RT SuperMix for qPCR Kit (Vazyme Medical Technology). The 2^−ΔCt^ method was used to process qPCR data. Primer sequences used were listed as follows: GAPDH-F, 5′-GGAGCGAGATCCCTCCAAAAT-3′, GAPDH-R, 5′-GGCTGTTGTCATACTTCTCATGG-3′; C1RL-AS1-F, 5′-GGCTCCCACTGATTCTACATTAGG-3′, C1RL-AS1-R, 5′-TCCTTCTCCTTCTACTCACAGAGC-3′; LINC00968-F, 5′- GCCCAGTTGACAGGAAATGT-3′, LINC00968-R, 5′-TTGGTTCTCAATGGGATGGT-3′; miR155HG-F, 5′-GAGTGCTGAAGGCTTGCTGT-3′, miR155HG-R, 5′-TTGAACATCCCAGTGACCAG-3′; CPB2-AS1-F, 5′-GCCTAGTAGGGGGACTTCCA-3′, CPB2-AS1-R, 5′-TCCATCCTTCCCCGCTAAGA-3′; LINC00906-F, 5′- CCGAAGAACCCTCAGTAGGC-3′, LINC00906-R, 5′-GGGTGGGCTATTGCTTGCTA-3′; WDR11-AS1-F, 5′-GACCCTTTGTCTGTTGCT-3′, WDR11-AS1-R, 5′- CAGAGGCCAGTCATCCTA-3.

### 2.14. Ethical approval statement

TCGA belongs to the public databases. The patients involved in the database have obtained ethical approval. Users can download data for free. Our study is based on this open-access database, so there are no ethical issues or other conflicts of interest.

## 3. Results

### 3.1. Construction of molecular typing based on TNF score-related lncRNAs

**Figure 2:**
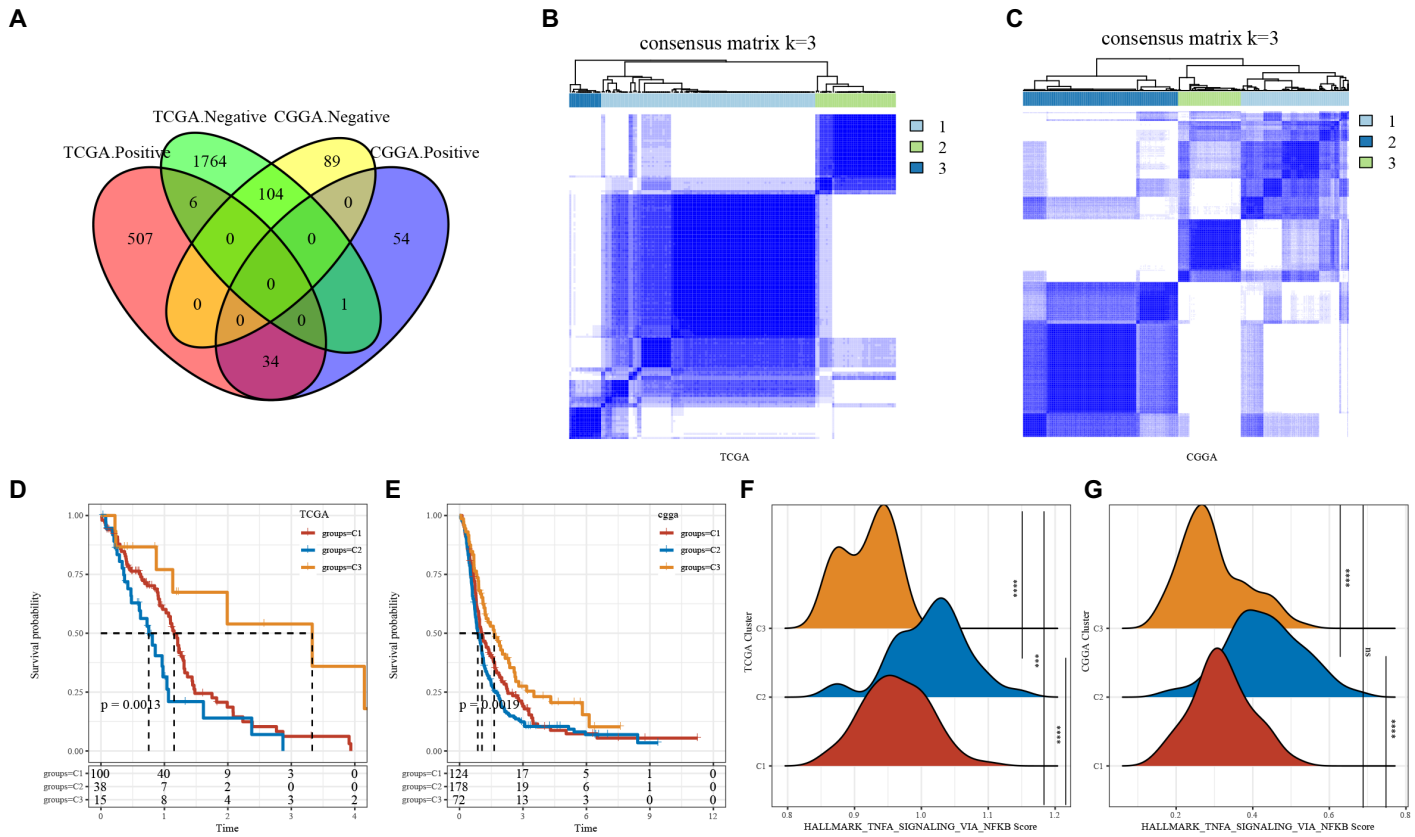
Tumor necrosis factor (TNF)-associated lncRNA subtypes in TCGA. **(A)** Venn diagram of the intersection between TNF activity-lncRNAs in the two cohorts. **(B)** Clustering heatmap of TCGA samples at consensus *k* = 3. **(C)** Clustering heatmap of CGGA samples at consensus *k* = 3. **(D)** Overall survival curve of TNF-related lncRNA subtypes of TCGA. **(E)** Overall survival curve of TNF-related lncRNA subtypes of CGGA; **(F)** TNF activity in TCGA cohort samples at three molecular subtypes; **(G)** TNF activity in CGA cohort samples at three molecular subtypes. ***P < 0.001, ****P < 0.0001.

First, by the correlation analysis of TNF activity scores and lncRNA expression, we identified 2,416 and 282 lncRNAs significantly associated with TNF activity in TCGA and CGGA, respectively, with 138 lncRNAs shown in Figure 2A intersection between them, suggesting poor consistency of TNF activity-associated lncRNAs between two datasets. Then, the GBM samples in the TCGA cohort were clustered by ConsensusClusterPlus, and the optimal number of clusters was determined based on the cumulative distribution function (CDF), from which the CDF Delta area curve showed that the clustering results were more stable when the cluster was selected as 3 subtypes. We ultimately chose *k* = 3 to obtain three molecular subtypes/clusters (Figure 2B). Further analysis of the prognostic characteristics of these three subtypes revealed significant prognostic differences, as shown in Figures 2D,E with an overall better prognosis for C3 and a worse prognosis for the C2 subtype. In addition, the same situation was seen in the CGGA cohort, as shown in Figures 2C,E. These results indicate that the three molecular subtypes of lncRNAs with TNF-related activity are transplantable in different research cohorts. In addition, we also compared whether there were differences in the TNF scores between the different molecular subtypes, as shown in Figures 2F,G. The TNF activity of the two subtypes in the TCGA and CGGA cohorts was significantly different, with a higher level of TNF activity in the C2 subtype and a lower TNF score in the C1 subtype.

### 3.2. Clinical characteristics among subgroups of TNF-related lncRNAs

In the TCGA dataset, we compared the distribution of different clinical features in the three molecular subtypes to determine whether the clinical features differed across subtypes and found IDH and TERT promoters in the TCGA dataset. The clinical characteristics differed significantly among subtypes (Supplementary Figures S2A–E). in the CGGA dataset, age, sex, IDH and 1p19q_codeletion clinical characteristics differed significantly among subtypes (Supplementary Figures S2F–I). There were more mutants of IDH in the C3 subtype in both datasets.

### 3.3. Comparison of differences In HALLMARK pathway scores between subtypes

**Figure 3:**
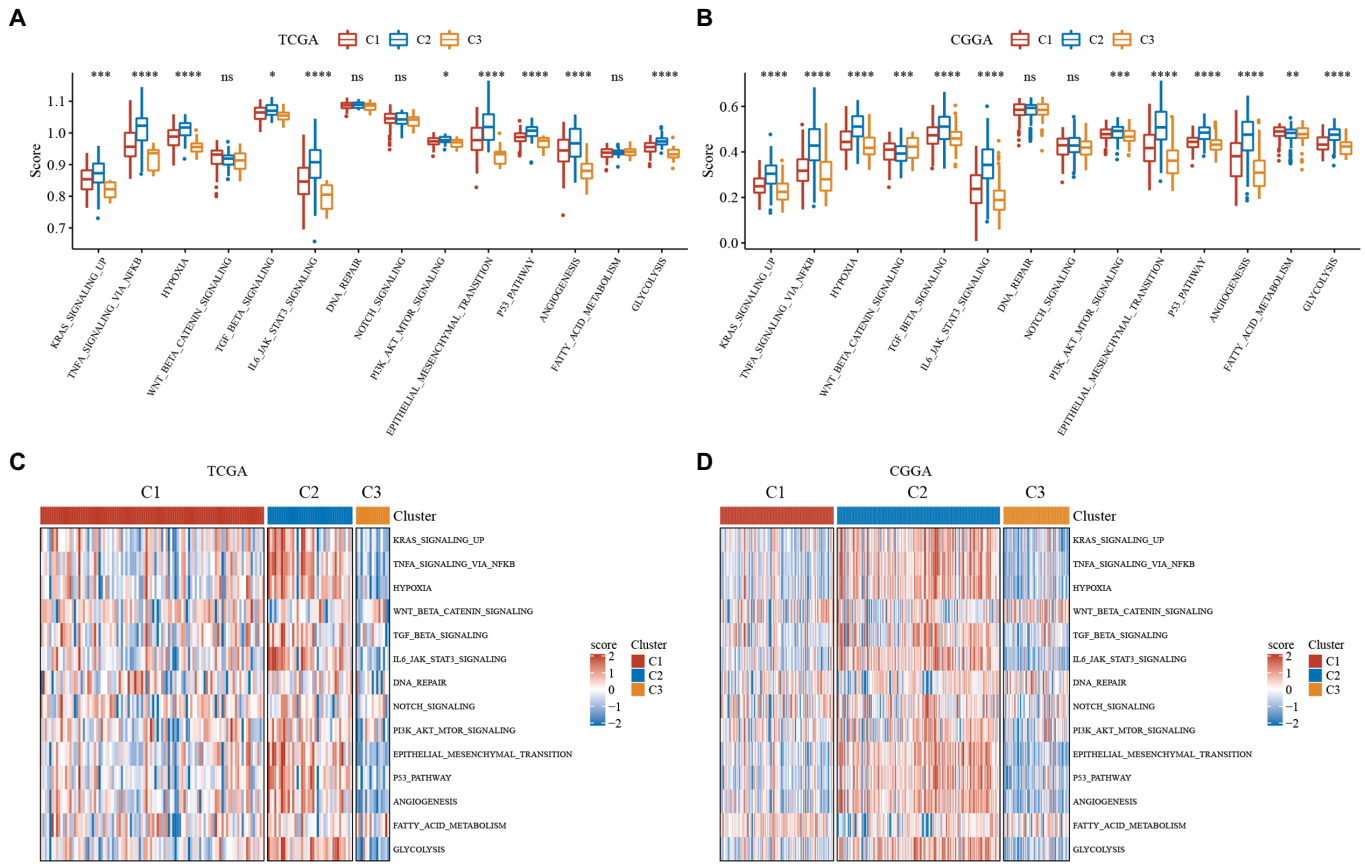
Single sample GSEA (ssGSEA) scores for hallmark molecular signatures related to tumor processes. Immune scores of ESTIMATE, MCP-counter of GBM. ABC, Differences in the distribution of immune microenvironment scores among subtypes in the TCGA dataset. DEF, Differences in the distribution of immune microenvironment scores among subtypes in the CGGA dataset. **(A)** Boxplot of the ssGSEA score of tumor-related pathways in the TCGA dataset. **(B)** Boxplot of the ssGSEA score of tumor-related pathways in the CGGA dataset. **(C)** Heatmap display of the ssGSEA scores of tumor-related pathways in the TCGA dataset. **(D)** Heatmap display of the ssGSEA scores of tumor-related pathways in the CGGA dataset. *P < 0.05, **P < 0.01, ***P < 0.001, ****P < 0.0001.

We used the ssGSEA method to score the TCGA and CGGA samples separately using HALLMARK and then compared tumor-related differences in TNFA signaling via NFkb, hypoxia, WNT beta-catenin signaling, TGF beta signaling, IL6 jak stat3 signaling and other pathway scores in subtypes. The results revealed that KRAS signaling up, TNFA signaling via NFkb and other pathways scores were significantly high in both TCGA and CGGA datasets in C2 subtypes with the worst prognosis, while the C3 subtype with the best prognosis had the scores were low in the best prognostic, while C1 subtype scores were in the middle (Figures 3A,B). We found that the C1 subtype may be in a transitional phase between C2 and C3. Thus, for a more visual presentation, we plotted this as a heatmap (Figures 3C,D).

### 3.4. Immunological characteristics of subtypes of TNF-related lncRNAs

To further elucidate differences in the immune microenvironment of patients in the subtype of TNF-related lncRNAs, we assessed the extent of immune cell infiltration of patients in our 3 GBM cohorts by the expression levels of gene markers in immune cells, where the marker genes in our immune cells were derived from the literature (25), and we used the software ESTIMATE and MCP-counter to evaluate the immune microenvironment.

**Figure 4:**
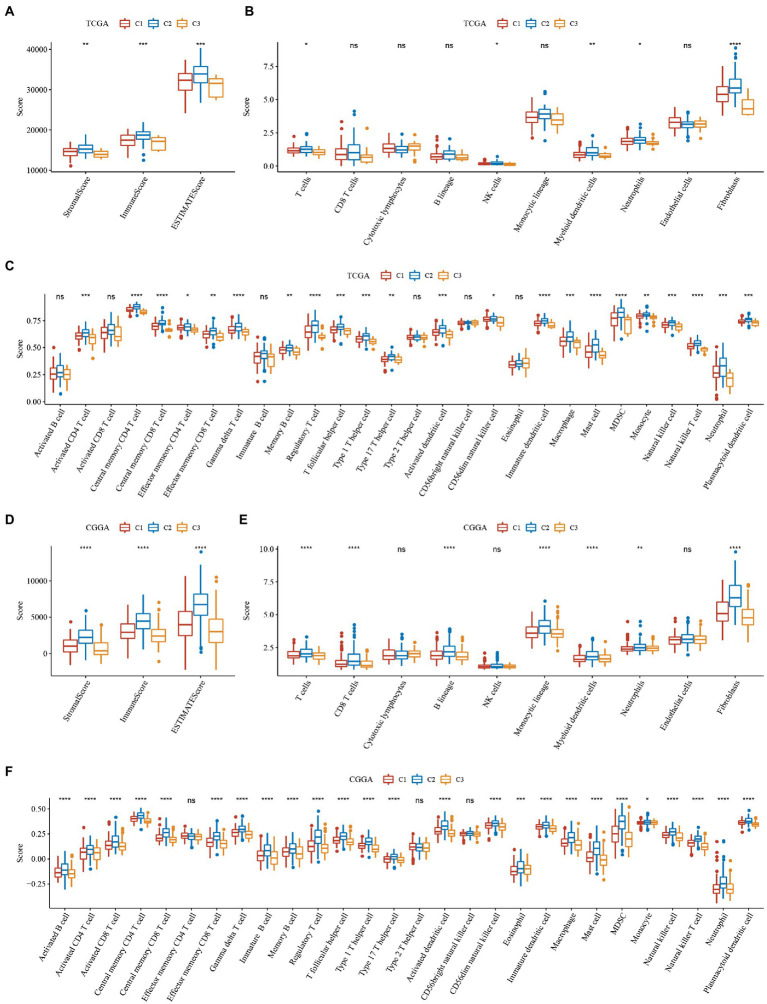
Immune scores of ESTIMATE, MCP-counter of GBM. **(A–C)** Differences in the distribution of immune microenvironment scores among subtypes in the TCGA dataset. **(D–F)** Differences in the distribution of immune microenvironment scores among subtypes in the CGGA dataset. *P < 0.05, **P < 0.01, ***P < 0.001, ****P < 0.0001.

The results of immune cells between different subtypes of TNF-related lncRNAs in the TCGA and CGGA cohorts are shown in Figure 4, where we can observe that the three molecular subtypes we defined differ significantly in most immune cells as well as in some pathways, as seen that: (1) Whether the immune scores of ESTIMATE, MCP-counter or ssGSEA. The C2 subtype with the worst prognosis had the highest score, while the C3 subtype with the best prognosis had the lowest immune score, and the C2 subtype with an intermediate prognosis had an intermediate middle immune score. This is consistent with the previously derived subtype analysis we did afore. The C1 subtype is in a transitional stage and belongs to the transitional subtype. (2) The distribution of immune infiltration is consistent in the TCGA and CGGA datasets, which indicates the stability of subtypes and the consistency of molecular characteristics.

### 3.5. Analysis of immune checkpoints

**Figure 5:**
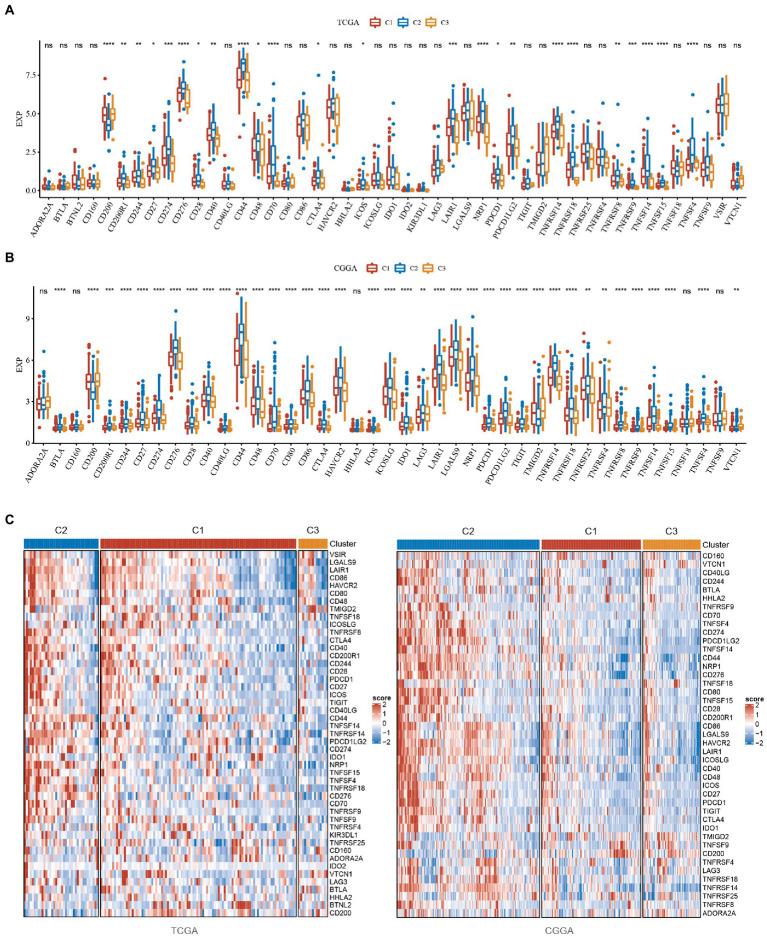
Analysis of immune checkpoints in GBM. **(A)** Differences in the expression distribution of immune checkpoints in the TCGA dataset among subtypes. **(B)** Differences in the expression distribution of immune checkpoints in the CGGA dataset among subtypes. **(C)** Differences in heatmap distribution of immune checkpoints in TCGA and CGGA datasets among subtypes. *P < 0.05, **P < 0.01, ***P < 0.001, ****P < 0.0001.

From the analysis of the above results, we know that the C2 subtype has the worst prognosis, as well as a high score of pathways associated with the same flow and the highest immune score among the three subtypes. The C3 subtype is the opposite of the C2 subtype, with the best prognosis, the lowest score of tumor-related pathways and the lowest immune score. The C2 subtype is in the intermediate stage and belongs to the transitional subtype. To explore why the C2 subtype had the worst prognosis but a high lack of immune score, we evaluated the distribution of expression of immune checkpoint and other genes in the subtypes. We found that: (1) 24 (51.06%) of the 47 immune checkpoints in TCGA were significantly different, and most of them were highly expressed in the C2 subtype with the worst prognosis (Figure 5A); (2) 38 (88.37%) of the 43 immune checkpoints in CGGA were significantly different and most of them are highly expressed in the C2 subtype with the poorest prognosis, which is consistent with the TCGA dataset (Figure 5B); (3) PDCD, CTLA4 and LAG3, which are associated with immune depletion, were significantly highly expressed in C2 subtype (Figure 5C), suggesting that immune depletion may occur in C2 which could explain why it has the highest immune score but the worst prognosis.

### 3.6. Analysis of chemokines

Chemokines have been found to play a key role in tumorigenesis and progression. They can mediate a variety of immune cells into the tumor microenvironment, helping T cells enter the tumor and influencing tumor immunity and therapeutic effects. Thus, we analyzed whether there was a differential expression distribution of chemokines among the 3 subtypes.

**Figure 6:**
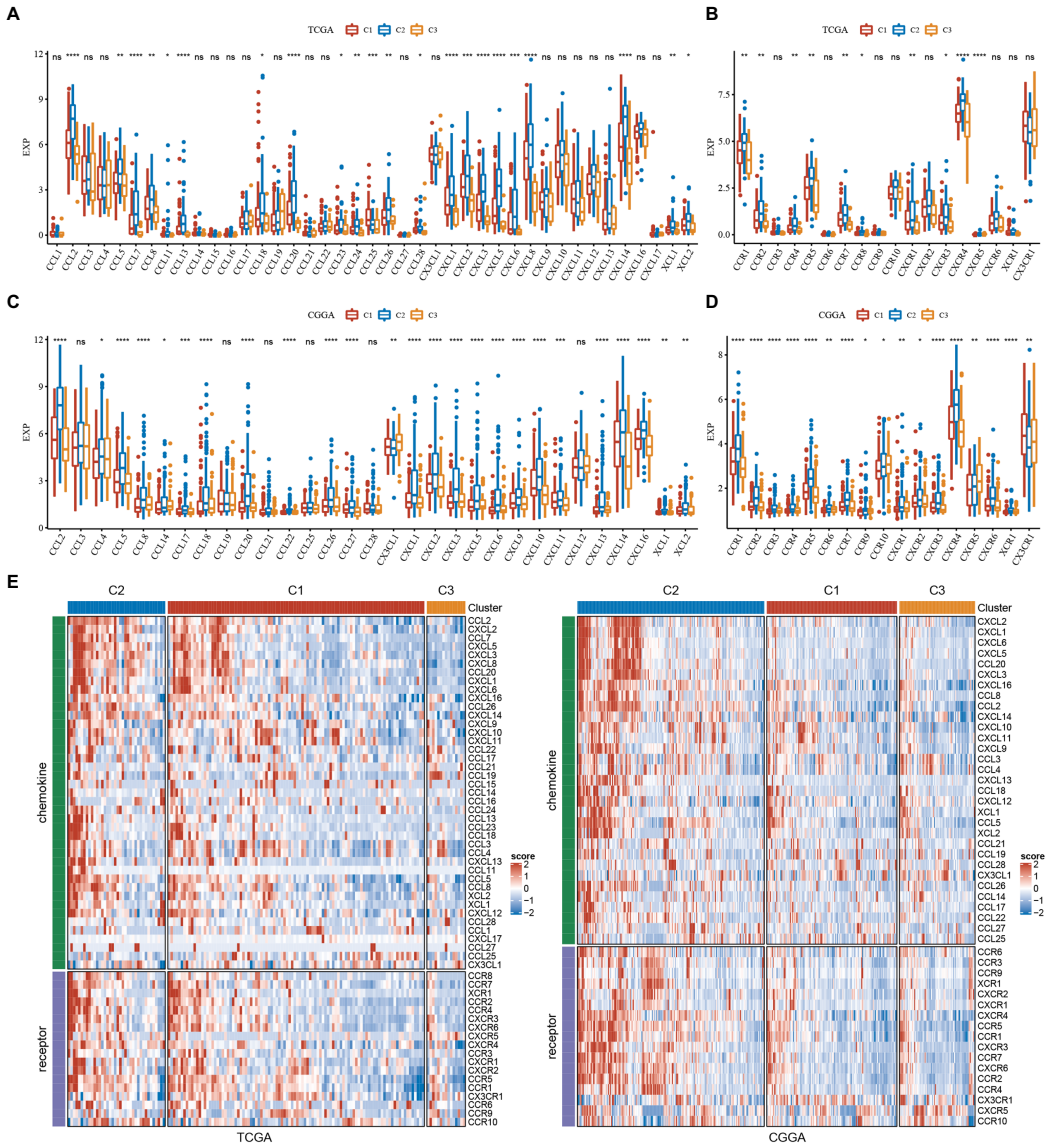
Analysis of chemokines and chemokine receptors in GBM. **(A)** Differences in the expression distribution of chemokines in TCGA. **(B)** Differences in the expression distribution of chemokine receptors in the TCGA cohort. **(C)** Differences in the expression distribution of chemokines in CGGA. **(D)** Differences in the expression distribution of chemokine receptors in the CGGA cohort. **(E)** Differences in the expression distribution of chemokines and receptors in the heatmaps among subtypes in TCGA and CGGA datasets. *P < 0.05, **P < 0.01, ***P < 0.001, ****P < 0.0001.

We calculated the differences in these genes in the TCGA cohort. As seen in Figures 6A, 22 of 41 (53.66%) chemokines differed significantly in subtypes, suggesting that the degree of immune cell infiltration may differ between subtypes and that these differences may contribute to differences in tumor progression and immunotherapeutic effects. In addition, we calculated and compared the expression of chemokine receptor genes in subtypes (Figure 6B) and found that 10 of 18 chemokine receptor genes were significantly differentially expressed among subtypes. By comparison, we calculated the differences in these genes in CGGA. Twenty-five of 31 (80.65%) chemokines differed significantly in subtypes (Figure 6C). This suggested that the degree of immune cell infiltration may differ among subtypes, which may contribute to differences in tumor progression and immunotherapy effects. In addition, we calculated and compared the expression of chemokine receptor genes in subtypes (Figure 6D) and found that 17 of 17 (100%) chemokine receptor genes were significantly differentially expressed between subtypes. The expression of chemokines and receptors remained consistent in the TCGA and CGGA subtypes, while both were highly expressed in the C2 subtype with the worst prognosis. The heatmap was performed to display them intuitively (Figure 6E).

### 3.7. Functional enrichment analysis of subtypes

**Figure 7:**
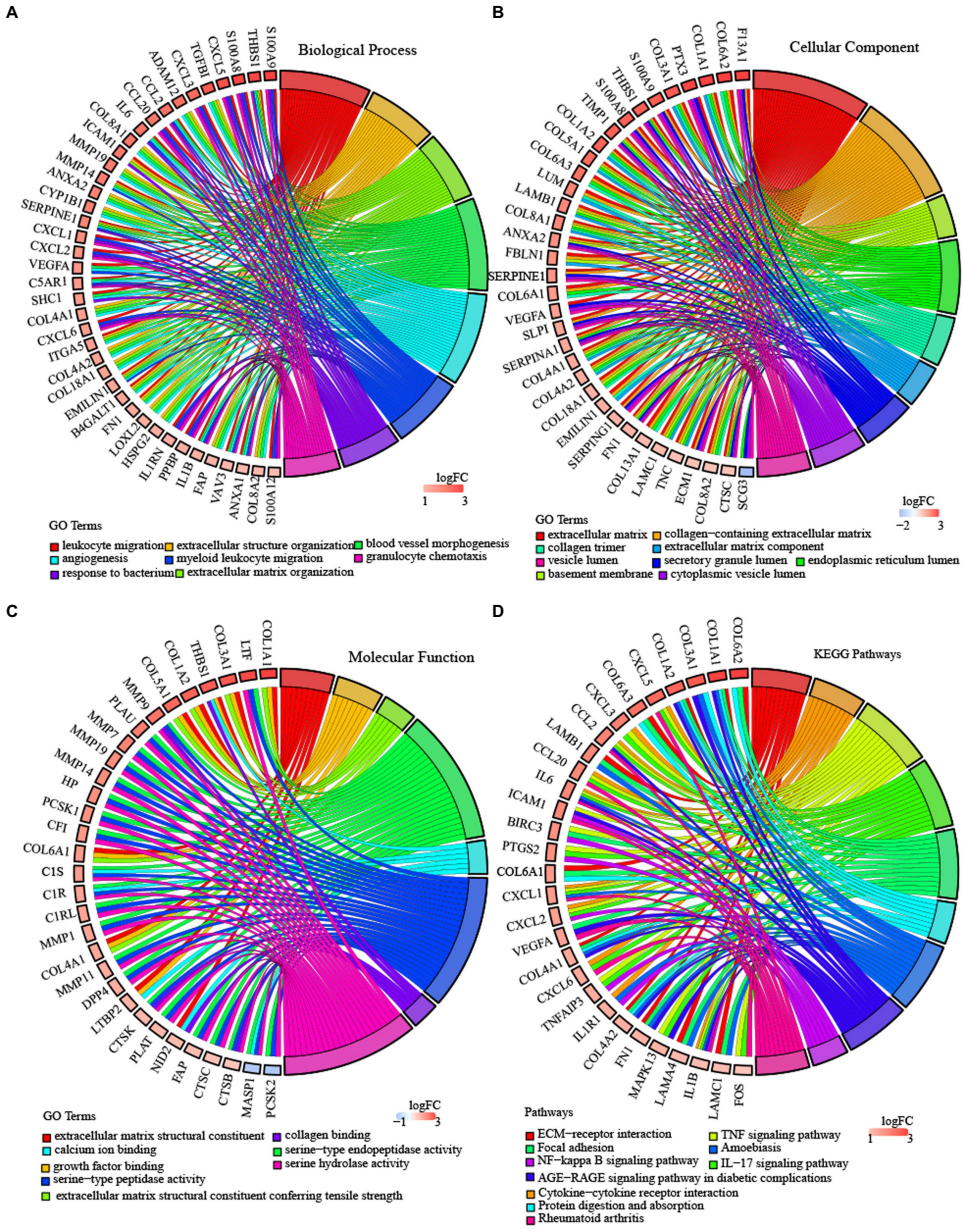
Enrichment analysis of subtypes in TCGA-GBM. **(A)** BP terms of differentially expressed genes in TCGA molecular subtype. **(B)** CC terms of differentially expressed genes in TCGA molecular subtype. **(C)** MF terms of differentially expressed genes in TCGA molecular subtype. **(D)** KEGG pathway analysis of differentially expressed genes in the TCGA molecular subtype. Validation of the expression level of 6 lncRNAs. *P < 0.05, **P < 0.01, ***P < 0.001, ****P < 0.0001.

To verify whether there are functional differences between these subtypes, we performed a differential analysis between C2 and C3, using limma to analyze their differential genes, and using criteria of |log2FC| > 1 and FDR <0.05, and finally get 815 differential genes, of which 517 genes were upregulated in C2, 298 genes were downregulated in C2 (Supplementary Datasheet S4). We then performed KEGG pathway analysis and Gene Ontology (GO) functional enrichment analysis on differentially expressed genes by WebGestaltR. For the GO functional annotations of differentially expressed genes, 488 items were annotated in BP with significant differences (FDR <0.05), and some of the annotation results are shown in Figure 7A. Seventy-five items were annotated in CC with significant differences (FDR <0.05), and some of the annotation results are shown in Figure 7B. Fifty-nine items were annotated in MF with significant differences (FDR <0.05), and some of the annotation results are shown in Figure 7C. Twenty-nine significant (FDR < 0.05) annotations for some differential gene KEGG pathway enrichment, as shown in Figure 7D, where ECM − receptor interaction, TNF signaling pathway, focal adhesion, cytokine−cytokine receptor interaction, NF − kappa B signaling pathway, IL − 17 signaling pathway and other tumors as well as immune-related pathways were also significant. The more detailed annotation is in Supplementary Datasheet S5.

To verify functional differences between subtypes, we performed a differential analysis between CGGA subtypes C2 and C3 using limma to analyze their differential genes and filtering using the criteria of |log2FC| > 1 and FDR <0.05. Finally, 951 differential genes were obtained, of which 532 were upregulated in C2 and 419 were downregulated in C2 (Supplementary Datasheet S6). We then performed KEGG pathway analysis and GO functional enrichment analysis on differentially expressed genes by WebGestaltR. For the GO functional annotations of differentially expressed genes, 433 items were annotated with significant differences (FDR <0.05) in BP, and some of the annotation results are shown in Supplementary Figure S3A. 117 items were annotated with significant differences (FDR <0.05) in CC, and some of the annotation results are shown in Supplementary Figure S3B. Eighty-four items were annotated with significant differences (FDR <0.05) in MF, and some annotation results are shown in Supplementary Figure S3C. Twenty-two items were annotated with significant differences (FDR <0.05) in KEGG pathways, and some of the annotation results are shown in Supplementary Figure S3D, where ECM − receptor interaction, TNF signaling pathway, focal adhesion and other tumor-related pathways were also significant. More details are shown in Supplementary Datasheet S7.

### 3.8. Comparison of assessment analysis of other characteristics

CD8+ T cells in the tumor microenvironment are capable of producing interferon-γ (IFNγ), which stimulates the upregulation of PD-1/PD-L1 and IDO1 gene expression (26, 27). It has been shown that the upregulation of IDO1 expression is positively associated with poor prognosis, tumor progression, and metastasis (28, 29). We extracted Th1/IFNγ signatures from previous research (30) and calculated IFNγ scores for each patient using the ssGSEA method. There was a significant difference in the score; C2 had the worst prognosis and the highest IFNγ score.

**Figure 8:**
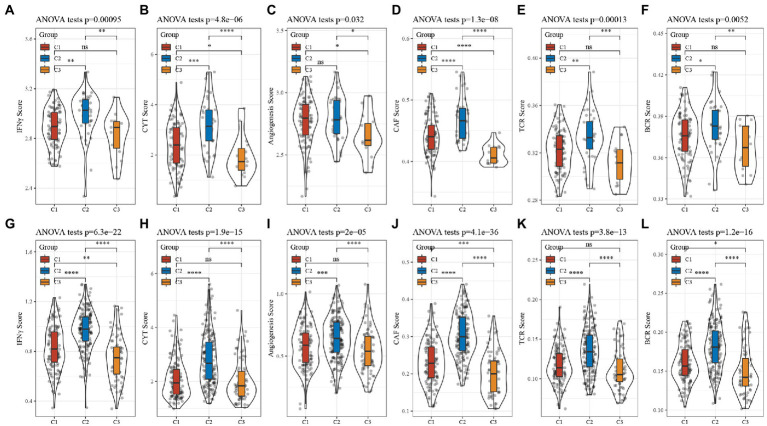
Analysis of other characteristics in GBM. **(A)** Differences in the distribution of IFNγ scores in the TCGA cohort. **(B)** Differences in the distribution of CYT scores in the TCGA cohort. **(C)** Differences in the distribution of angiogenesis scores in the TCGA cohort. **(D)** Differences in the distribution of CAF scores in the TCGA cohort. **(E)** Differences in the distribution of TCR scores in the TCGA cohort. **(F)** Differences in the distribution of BCR scores in the TCGA cohort. **(G)** Differences in the distribution of IFNγ scores in the CGGA cohort. **(H)** Differences in the distribution of CYT scores in the CGGA cohort. **(I)** Differences in the distribution of angiogenesis scores in the CGGA cohort. **(J)** Differences in the distribution of CAF scores in the CGGA cohort. **(K)** Differences in the distribution of TCR scores in the CGGA cohort. **(L)** Differences in the distribution of BCR scores in the CGGA cohort.

According to the previous Rooney Michael S, the average evaluation of GZMA and PRF1 expression levels was used to assess the intertumoral immune T cell lysis activity of each patient (31). There were significant differences in the three subtypes, as shown in Figure 7, with C2 having the worst prognosis and the highest score. The angiogenesis-related gene set was obtained from previous research to assess the angiogenesis scores of each patient (32). Significant differences in different subtypes, as shown in Figure 8, with higher scores for C2. In addition, we evaluated cancer associated fibroblast (CAF) (33), B-cell receptor (BCR) (34), and T-cell receptor (TCR) (34) scores and found that they all had the highest scores in the C2 subtype with the worst prognosis. The distribution of these six scores remained consistent in the TCGA and CGGA subtypes.

### 3.9. Gene set enrichment analysis pathway enrichment analysis of subtypes

To investigate the pathways of different molecular subtypes in biological processes, we used GSEA for pathway analysis, where we used all candidate gene sets in the KEGG database (24) for subtypes C2 and C3 to conduct gene set enrichment analysis (GSEA). Then, filtered using *p* < 0.05 and FDR < 0.25, it showed that: (1) The ECM_RECEPTOR_INTERACTION, JAK_STAT_SIGNALING_PATHWAY, VEGF_SIGNALING_PATHWAY, FOCAL_ADHESION and other pathways related to tumors in the C2 subtype in the TCGA dataset are significantly enriched, and the immune-related T_CELL_RECEPTOR_SIGNALING_PATHTOWAY, NATURAL_TOTORYINTERALING_TOYTOW and NATURAL_KILLER_PATH_TOYTO were also significantly enriched; (2) The enrichment results in the CGGA dataset are consistent with the results of TCGA, with tumor as well as immune-related pathways were significantly enriched in C2 subtypes, Supplementary Figure S4.

### 3.10. Identification of and enrichment analysis of TNF-related lncRNAs in GBM

**Figure 9:**
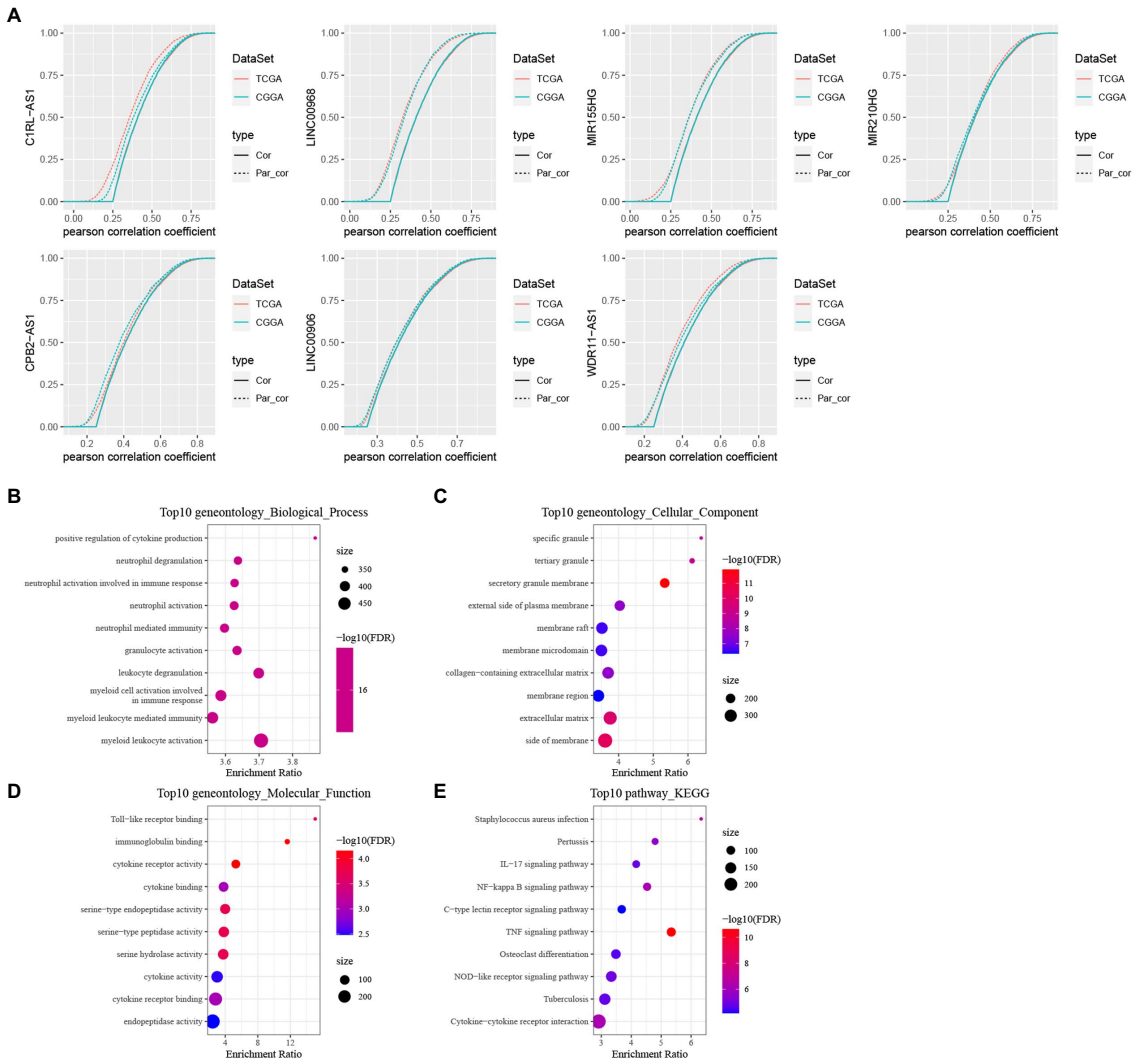
Identification of TNF- and construction of these six lncRNAs signature. **(A)** Cumulative distribution curve with or without adjustment for lncRNA by using a first order partial correlation. Solid lines indicate CDFs of correlation coefficients between TNF scores and gene expressions without adjustment. Dashed lines indicate first order partial correlation adjusted relations between TNF scores and gene expressions. These two distributions were compared using the Kolmogorov Smirnov test. The x axis represents Pearson correlation coefficients between glycolysis scores and gene expressions, and the *y* axis represents cumulative probabilities. **(B–E)** KEGG pathway analysis and GO functional enrichment analysis of lncRNA-related PCGs.

Based on univariate cox of 138 lncRNAs were obtained, which were related to prognosis with GBM in both TCGA and CGGA cohorts and then we performed the first-order partial correlation analysis by TNF score, TNF-related genes and lncRNAs expression levels and when the influence of these six lncRNAs was removed effects, the correlation between TNF scores and TNF-related genes decreased significantly. Finally, we identified six key lncRNAs: C1RL-AS1, LINC00968, MIR155HG, CPB2-AS1, LINC00906, and WDR11-AS1 (Figure 9A).

We calculated the Pearson correlation between lncRNAs and PCGs in TCGA and CGGA, respectively, and then obtained the PCGs positively correlated with lncRNA according to Corr >0.4 and *p* < 0.05, and then took the intersection of positively correlated genes from different data sets to obtain a total of 646 PCGs, and then use the R package WebGestaltR to perform KEGG pathway analysis and GO function enrichment analysis on the differential genes (Figures 9B–E).

### 3.11. Predictive signature construction and validation

**Figure 10:**
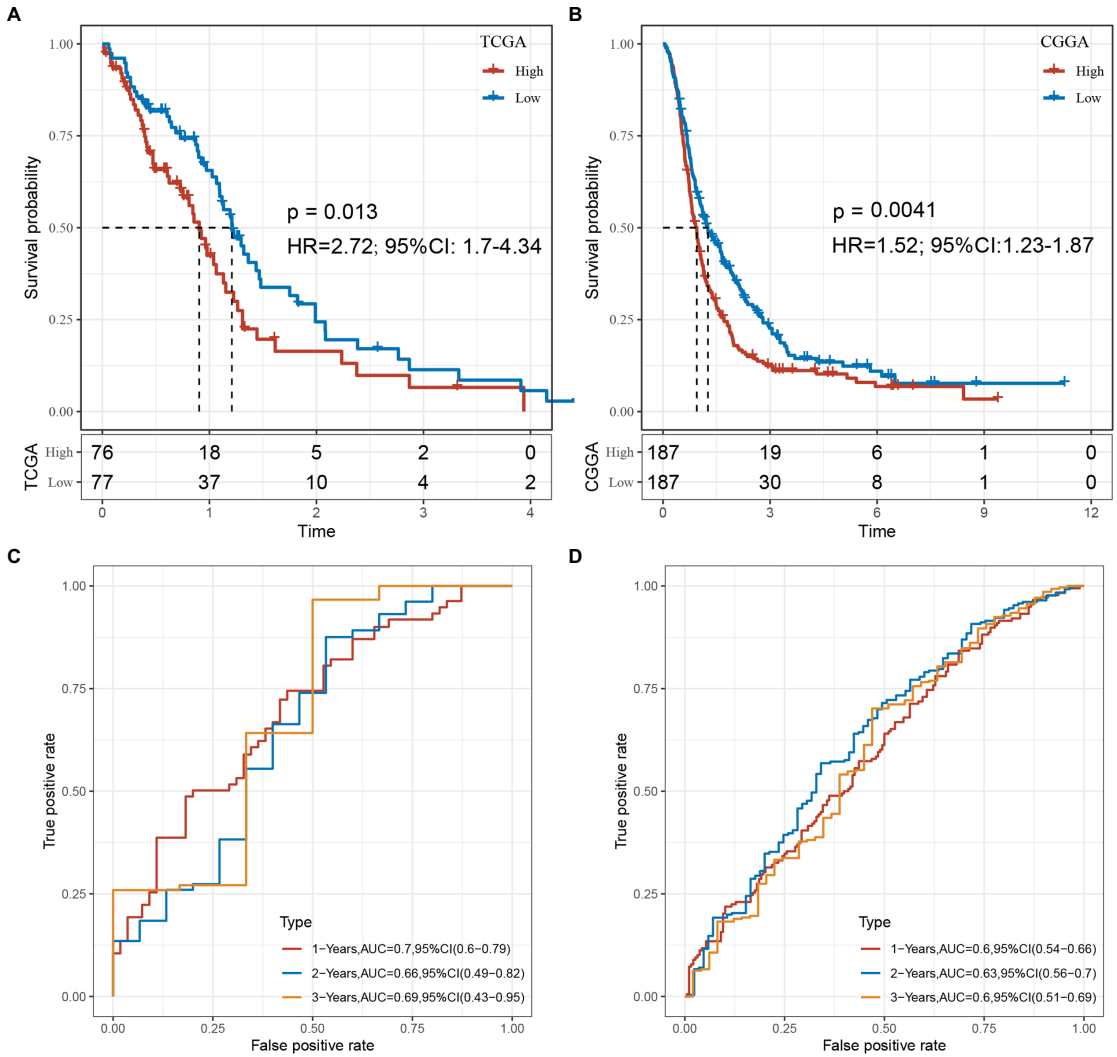
**(A)** KM curve of the high- and low-risk groups in the TCGA cohort. **(B)** KM curve of the high- and low-risk groups in the CGGA cohort. **(C)** The 1, 2, 3-year ROC of TCGA-GBM cohort. **(D)** The 1, 2, 3-year ROC of CGGA-GBM cohort.

Based on these 6 lncRNAs, we performed the multivariate analysis in TCGA to calculate the risk value of each sample and divided the samples into high and low risk groups based on the median value the result was showed in Supplementary Figure S5, while validating them in CGGA, we could find significant differences in the prognosis of high and low risk groups in the two GBM datasets, suggesting that these 6 lncRNAs we identified can reflect predicted prognosis in GBM patients (Figures 10A,B). The risk score was calculated as follows: Risk Score = 0.384125 * EXP_C1RL-AS1_ + 0.303024 * EXP_LINC00968_ + (−0.00621) * EXP_MIR155HG_ + (−0.0647) * EXP_CPB2-AS1_ + (−0.40509) * EXP_LINC00906_ + (−0.1972) * EXP_WDR11-AS1_.

Combine the with clinical characters construct the multivariate cox regression analysis in both TCGA and CGGA (Supplementary Figures S6, S7). In order to evaluate whether our 6-lncRNAs signature has stable predictive performance, the validation of the nomogram in predicting the overall survival of GBM in the TCGA and CGGA dataset was shown in Supplementary Figure S8. The ROC of 1, 2, and 3 years data were analyzed in TCGA-GBM and CGGA-GBM cohorts. The AUC of our model in 1-year was 0.7, 2-years was 0.66, 3-years was 0.69 in TCGA-GBM, such as Figure 10C. The AUC of our model in 1-year was 0.6, 2-years was 0.63, 3-years was 0.6 in CGGA-GBM, such as Figure 10D. These results suggested that our study provides a good predictive model for predicting OS in patients with GBM.

### 3.12. Expression level of TNF related-lncRNAs in glioma and astroglia cells

**Figure 11:**
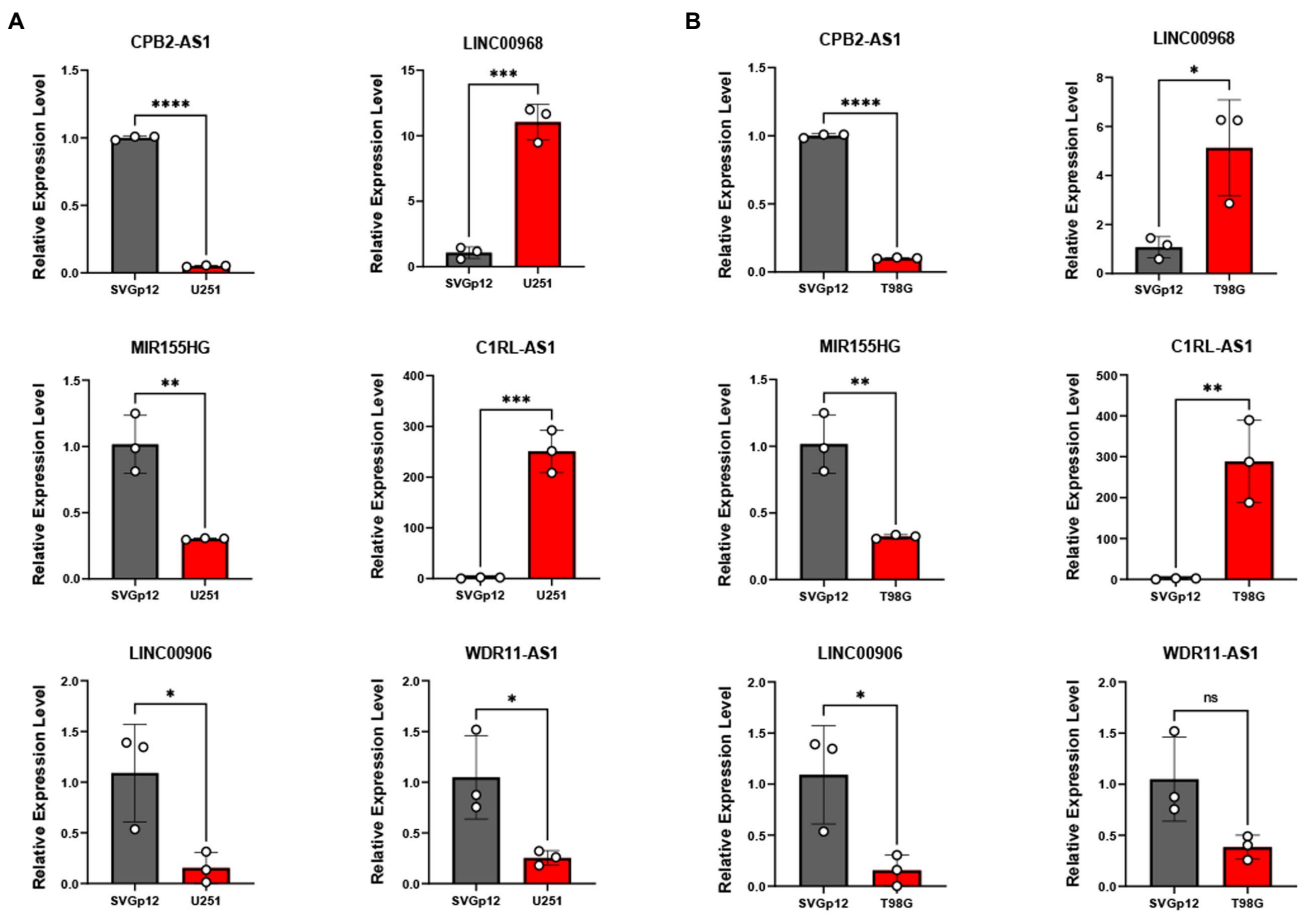
qRT-PCR was applied to measure these six TNF-related lncRNAs expression between U251 **(A)**, T98G **(B)**, and SVGp12. *P < 0.05, **P < 0.01, ***P < 0.001, ****P < 0.0001.

Furthermore, we examined the expression of the signature lncRNAs in cell lines U251 and T98G as tumor group, SVGp12 as normal or control group, by qPCR (Figure 11) analysis, showing that the expression of CPB2-AS1, MIR155HG, LINC00906 and WDR11-AS1 were low in gliomas cells. However, expression of LINC00968 and C1RL-AS1 were high in glioma cells. These results showed that the expression level of signature lncRNAs consistent with the Risk Score.

## 4. Discussion

Gliomas, especially glioblastomas, are the most devastating brain tumors in the human nervous system. In recent years, although advances have been made in the diagnosis and treatment of gliomas, the infiltrative and rapidly proliferating nature of gliomas makes it difficult to cure the tumor only by surgical treatment alone, and the prognosis of patients with postoperative recurrence is very poor, with the median survival time extended by only a few months (35, 36). The complexity of glioma is mainly reflected through its molecular heterogeneity. In recent years, molecular features have been recognized as key determinants of prognosis and treatment response in GBM (37, 38) Molecular subtypes can well predict the occurrence and development of glioma polymorphisms and can help us guide scientific treatment strategies (37).

The tumor immune microenvironment plays an important role in tumorigenesis and progression, it can affect treatment response (39, 40). In this study, we first identified TNF-related lncRNAs and constructed TNF-related lncRNA biomarkers that were stable enough to classify TCGA-GBM and CGGA-GBM cohorts into three molecular subtypes, with the C2 subtype having the worst prognosis and the C3 subtype having the best prognosis. Our data showed that immune infiltration macrophages were significantly highly expressed in subtypes with high TNF scores. This result is consistent with previous results, which indicate that immune infiltrating macrophages are associated with a high risk of disease and poor overall survival (41).

Our data showed that immune exhaustion-related PDCD1, CTLA4, and LAG3 are significantly highly expressed in subtypes with high TNF scores, that PD-L1 and PD-L2 can inhibit the activation and cytotoxic effects by binding to PD-1 and that CD80 and CD86 bind to CTLA4 and inhibit the T cell costimulatory signaling pathway. Thus, we speculate that tumors of the C2 subtype are in a state of “high immune infiltration with low immune response,” suggesting that immune exhaustion may occur in the C2 subtype. Therefore, C2 has the highest immune score but the prognosis. These findings suggested an important role in immune regulation and therapeutic resistance, both of which involve complex interactions of multiple pathways and components. We consistently observed a highly significant enrichment of tumorigenesis-related pathways and markers in the C2 molecular subtype with high TNF scores, such as the ECM receptor interaction, JAK Stat signaling pathway, VEGF signaling pathway, and focal adhesion pathways, which were significantly enriched, while immune-related T cell receptor signaling pathway, natural killer cell mediated cytotoxicity, and toll-like receptor signaling pathway were similarly significantly enriched, and these results tentatively revealed the mechanism for poor prognosis in the high-risk group. Even after controlling for the major confounding factors, these six TNF-related lncRNAs can still independently predict prognosis and immunotherapy response, highlighting their potential as individualized treatment-guiding biomarkers. Therefore, our research may contribute to the understanding of the role of TNF in cancer progression and antitumor immune responses, providing important insights for more effective immunotherapeutic strategies. Some of the TNF-related lncRNAs included in our prognostic model have previously been reported to play an important role in cancer progression. For example, in the current studies, lncRNA C1RL-AS1 and MIR210HG were both associated with poor prognosis in gastric cancer (42, 43). LINC00968 inhibits the proliferation, migration and invasion of lung adenocarcinoma (44) Our study identified the prognostic lncRNAs that TNF-related might target, thereby providing novel insight into their potential roles in the progression of GBM.

With the development of NGS research around the world, the classification of gliomas at the molecular level has made great progress. In the latest WHO classification of gliomas, many emerging molecular markers, such as IDH mutation status and 1p/19q codeletion status, have been incorporated, greatly facilitating the individualized treatment of glioma patients. However, the current classification still cannot fully describe the heterogeneity of gliomas. For example, IDH mutation status is an extremely critical molecular marker for glioma, and the overall prognosis of IDH wild-type patients is significantly worse than that of IDH mutant patients. Patients with IDH wild-type, even at WHO Grade I or II, are still considered to have a poor prognosis. There is even a view that most IDH wild-type gliomas are actually misdiagnosed (45). However, there is also great heterogeneity among patients with IDH-mutant gliomas (46). However, some problems associated with traditional standard treatment regimens, such as decreased neurological function, cannot be ignored. There is still a need to develop new strategies to improve outcomes and side effects, and a new perspective is still needed to further differentiate patients and develop more individualized treatment plans for patients to improve patient benefit. There were many clinical studies have focused on molecular markers, and their results indicate that molecular markers have good application prospects in the diagnosis, treatment of glioma and prognosis prediction (47–49). On the one hand, it is easy to see that the six-lncRNA model that we built each of them had a weak prognostic effect, but the risk score constructed by the combination had a strong ability to differentiate prognosis for GBM in both the TCGA and CGGA cohorts. Multivariate analysis also confirmed the independence of this model. This study may provide a new perspective for the risk assessment of GBM patients and a new complement to the risk stratification of GBM patients. Immune checkpoint blockade therapy is an emerging antitumor therapy in recent years that targets and blocks suppressive immune checkpoints in tumors, such as CTLA4 and PD1, and their ligands PD-L1 and CD86 in tumors to break the negative feedback inhibition of immune checkpoints against tumor immunity, thereby inducing the immune system to kill tumors, and has achieved excellent therapeutic effects in a variety of cancers (50). However, immune checkpoint blockade therapy is not effective in all tumors, as the effectiveness of immune checkpoint blockade therapy is predicated on the presence of CD8 T cell infiltration and its suppression by immune checkpoints (51). Our results showed that the high-risk group defined by our risk model had a relatively higher CD8 T cell infiltration level, which is consistent with the “high immune infiltration, low immune response” characteristics for immune checkpoint therapy to be effective, suggesting that immune checkpoint blockade therapy may be a good complementary therapy.

## 5. Conclusion

In summary, we molecularly typed two paired GBM cohorts (TCGA and CGGA) based on TNF-related lncRNAs, and each subtype showed significant differences in prognosis. Different molecular types have a different clinical, mutational, pathway and immunological characteristics. Six key lncRNAs of TNF-related pathways were also identified, and a prognostic model was constructed. The model scores had a strong prognostic value and ability to predict the immunotherapeutic response in GBM patients. This study provides new insights to guide more effective treatment strategies.

## Data availability statement

The original contributions presented in the study are included in the article/Supplementary material, further inquiries can be directed to the corresponding authors.

## Author contributions

XL and WW decided the conception and design of general outline, and finally completed the study supervision. SRL, BBW, and LY performed the experiments, analyzed the data and revised the manuscript. LSW, BW, and YY: prepared the figures and tables. JJZ, BY, QQZ, MS, LY, and LW drafted the work and collected the materials. All authors contributed to the article and approved the submitted version.

## Funding

This work was supported by grants from the National Natural Science Foundation of China (No. 82001421).

## Conflict of interest

The authors declare that the research was conducted in the absence of any commercial or financial relationships that could be construed as a potential conflict of interest.

## Publisher’s note

All claims expressed in this article are solely those of the authors and do not necessarily represent those of their affiliated organizations, or those of the publisher, the editors and the reviewers. Any product that may be evaluated in this article, or claim that may be made by its manufacturer, is not guaranteed or endorsed by the publisher.

## Footnotes

^1^ https://portal.gdc.cancer.gov/

^2^ http://www.cgga.org.cn/

^3^ https://www.gencodegenes.org/
