# Supplementary figures and images for: Novel tumor necrosis factor-related long non-coding RNAs signature for risk stratification and prognosis in glioblastoma
Source: Front Neurol. 2023 Apr 20;14:1054686. doi: 10.3389/fneur.2023.1054686 (PMC10156969; doi:10.3389/fneur.2023.1054686)

A

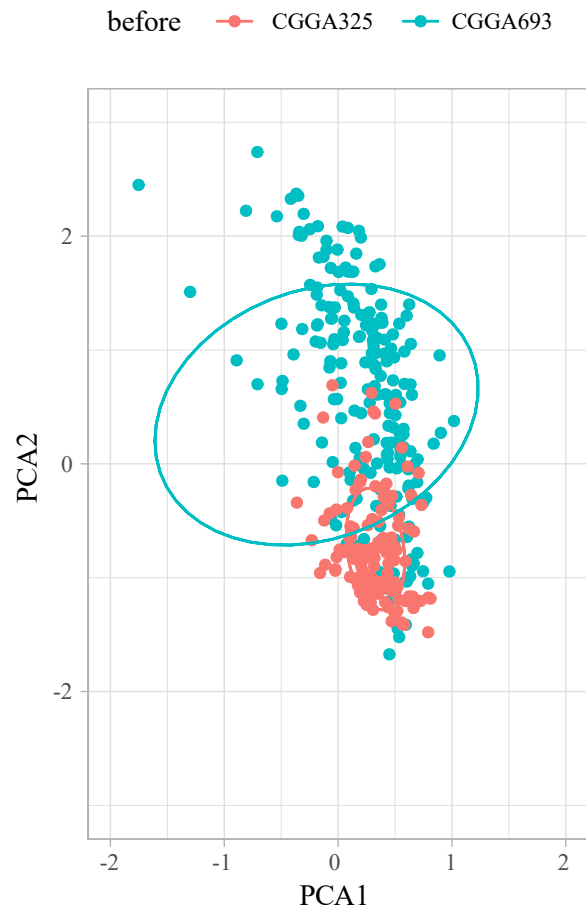

B

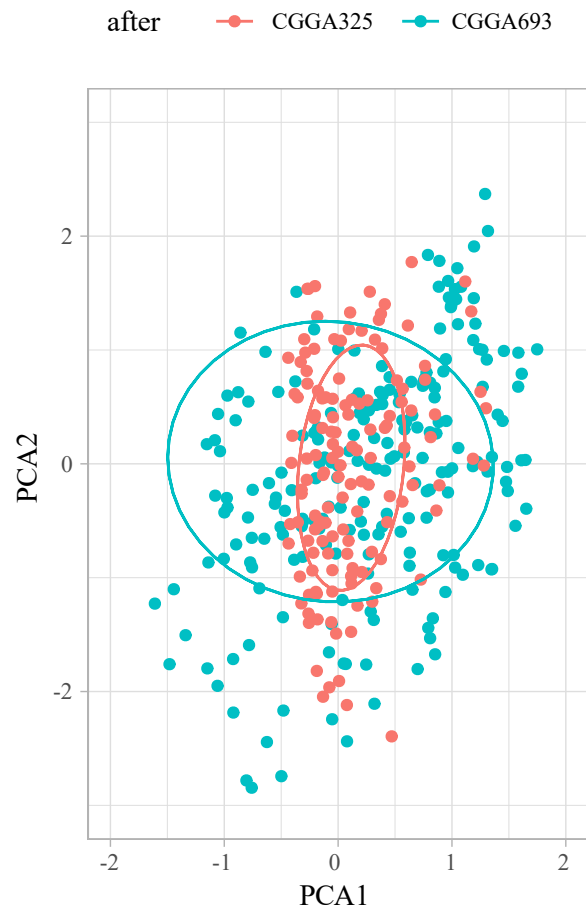

Supplement: Supplementary file 9 [file Image_1.PDF]

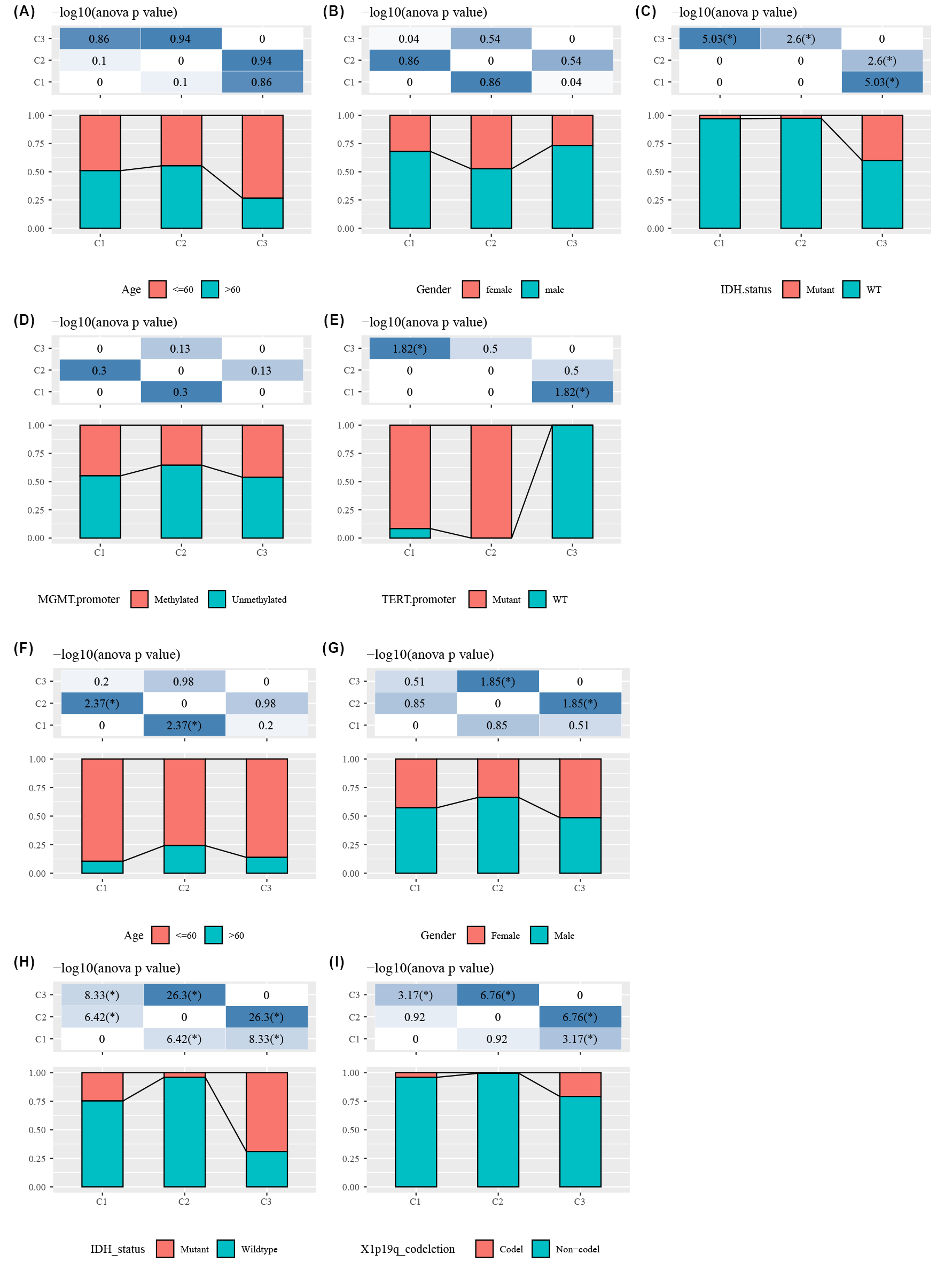

Supplement: Supplementary file 10 [file Image_2.TIF]

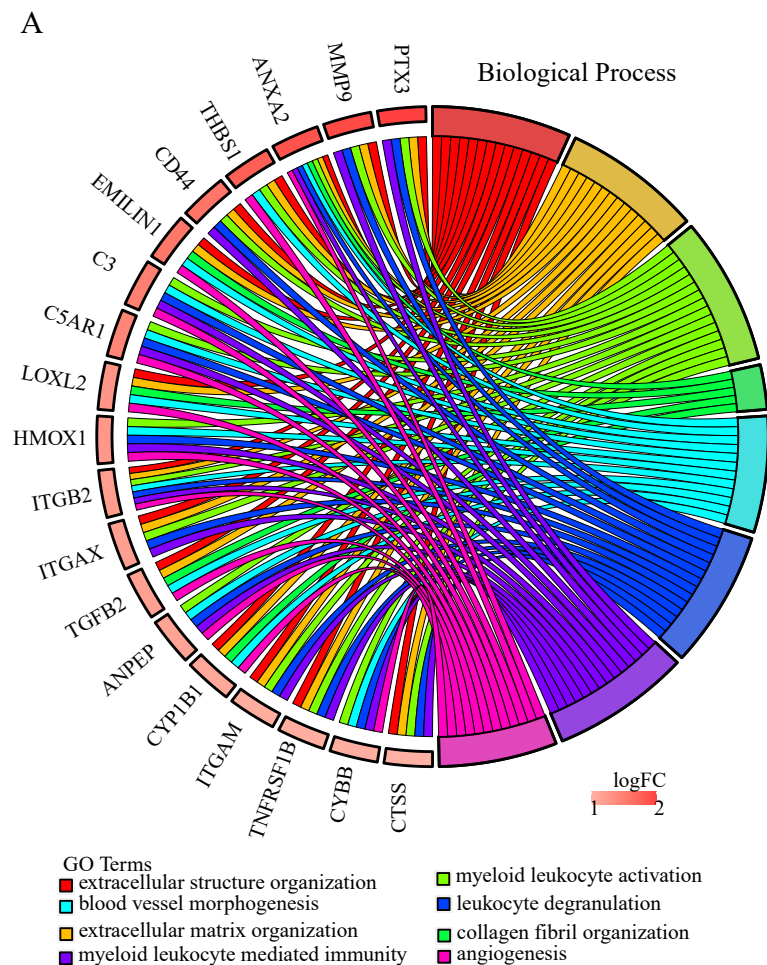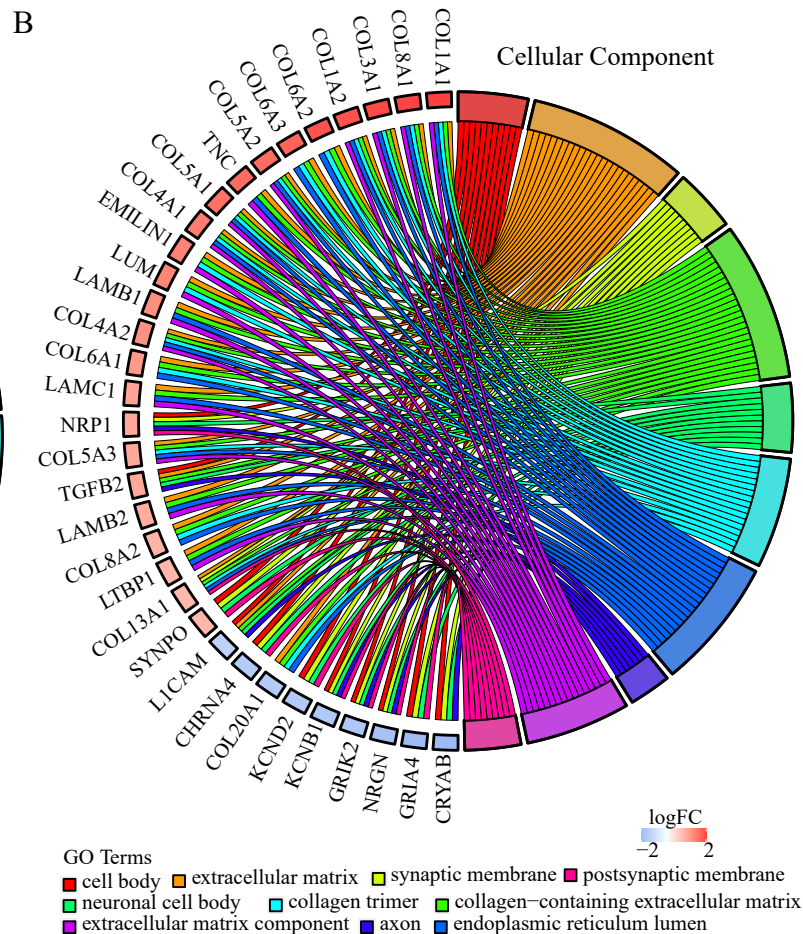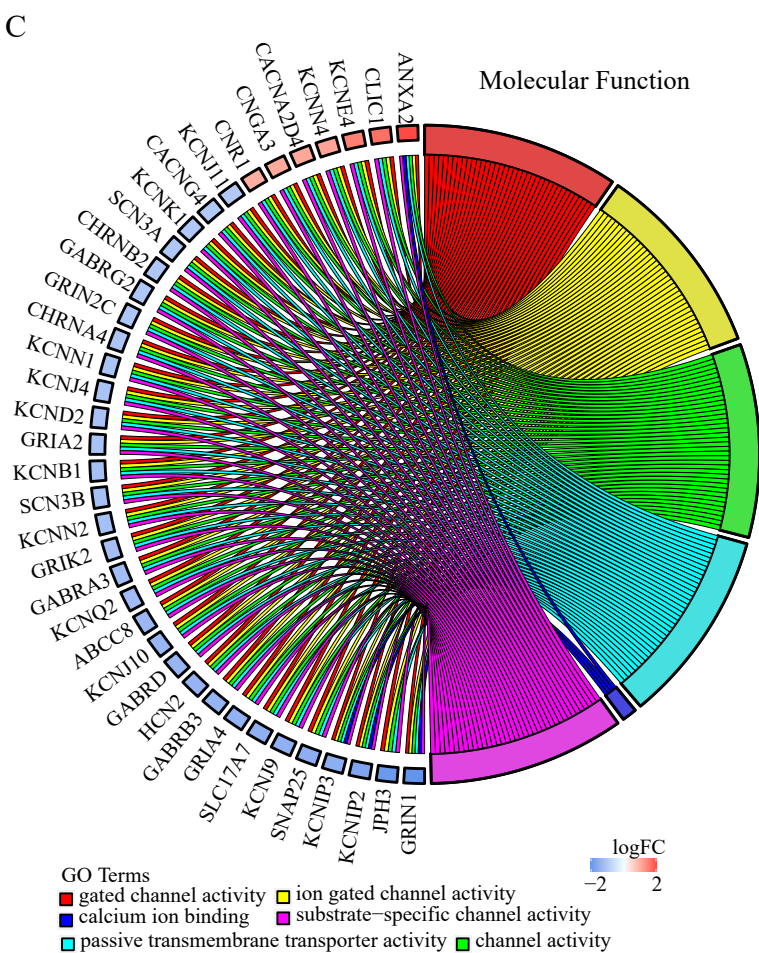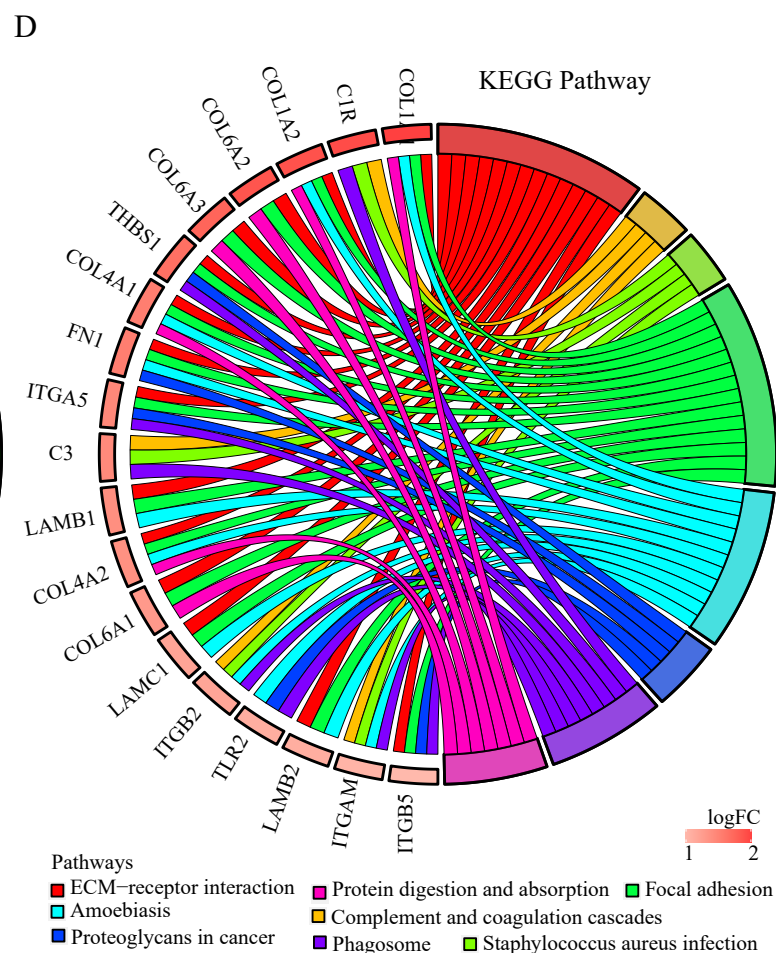

Supplement: Supplementary file 11 [file Image_3.PDF]

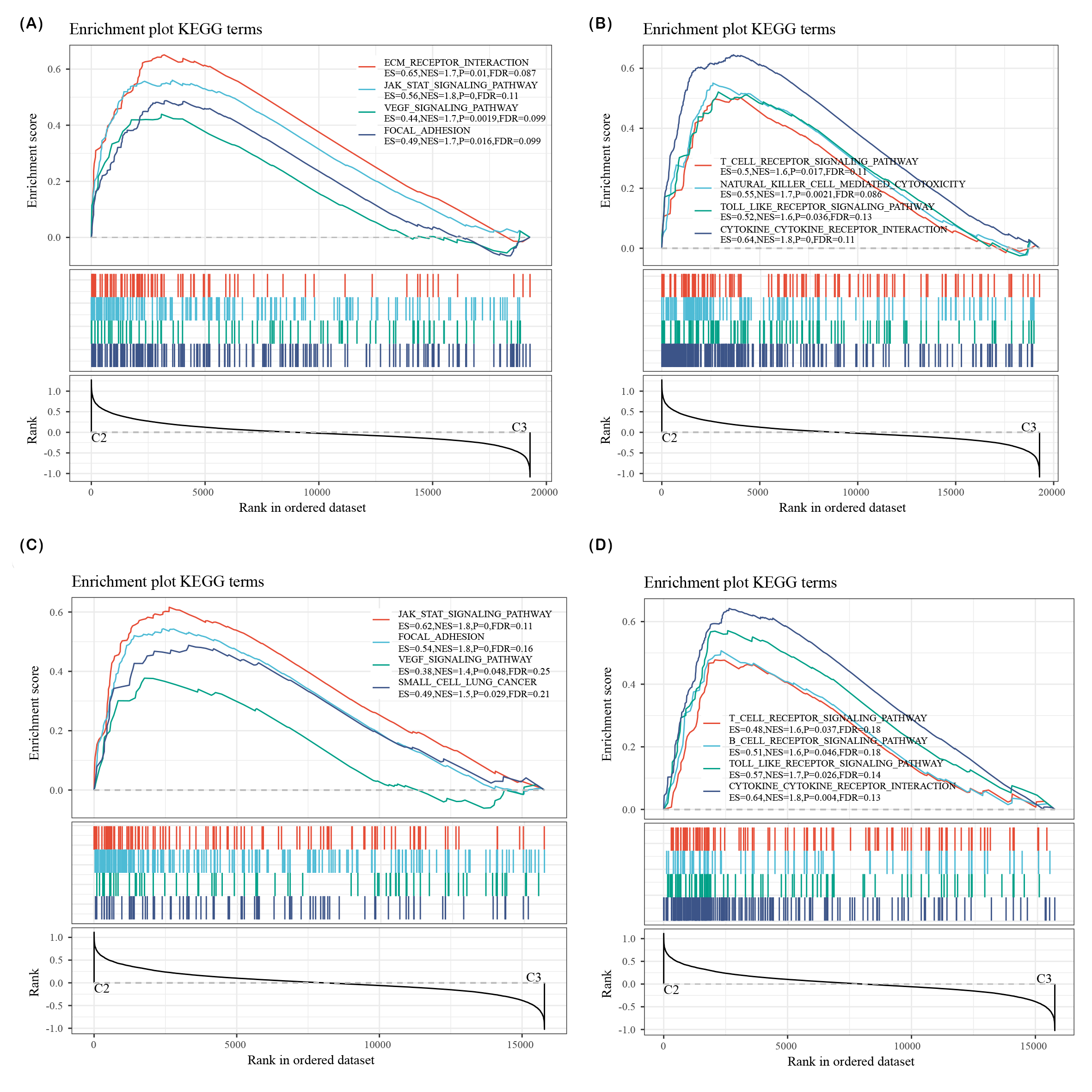

Supplement: Supplementary file 12 [file Image_4.TIF]

# Hazard ratio

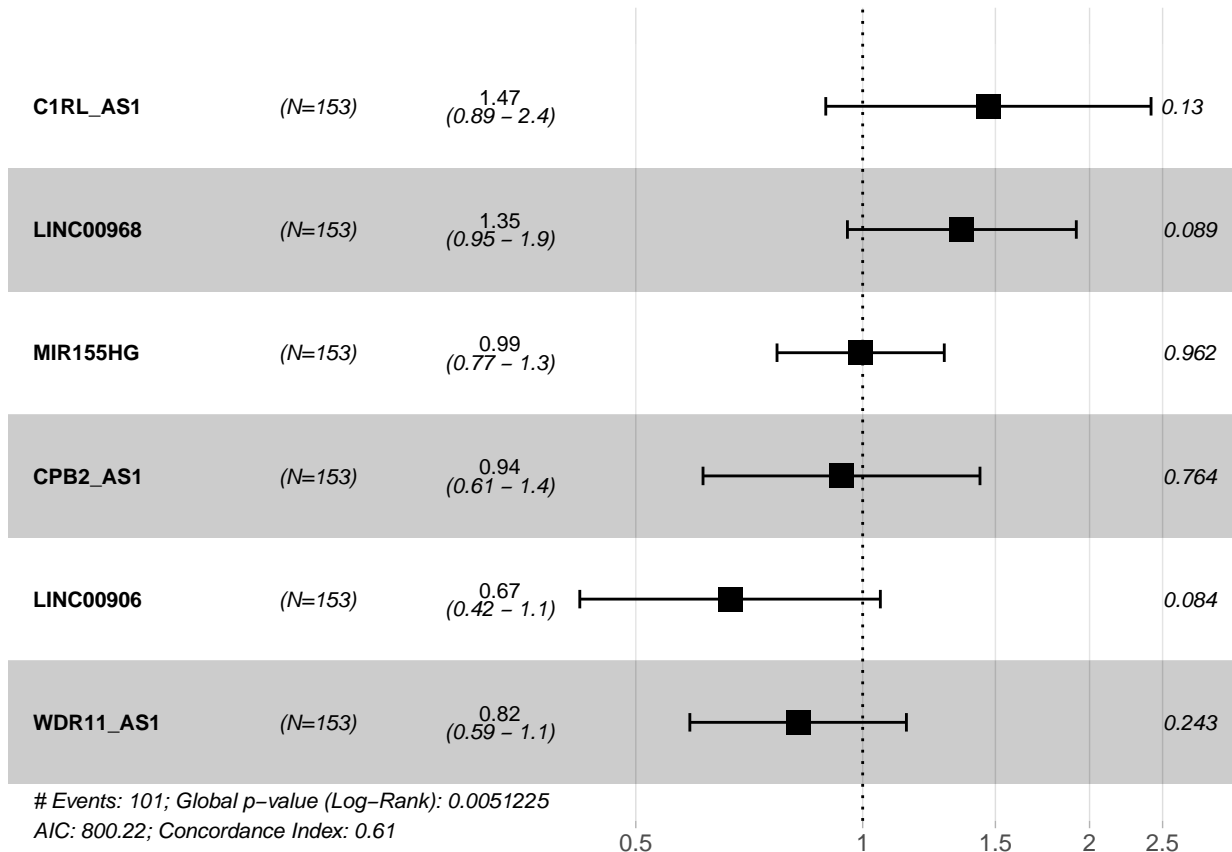

Supplement: Supplementary file 13 [file Image_5.PDF]

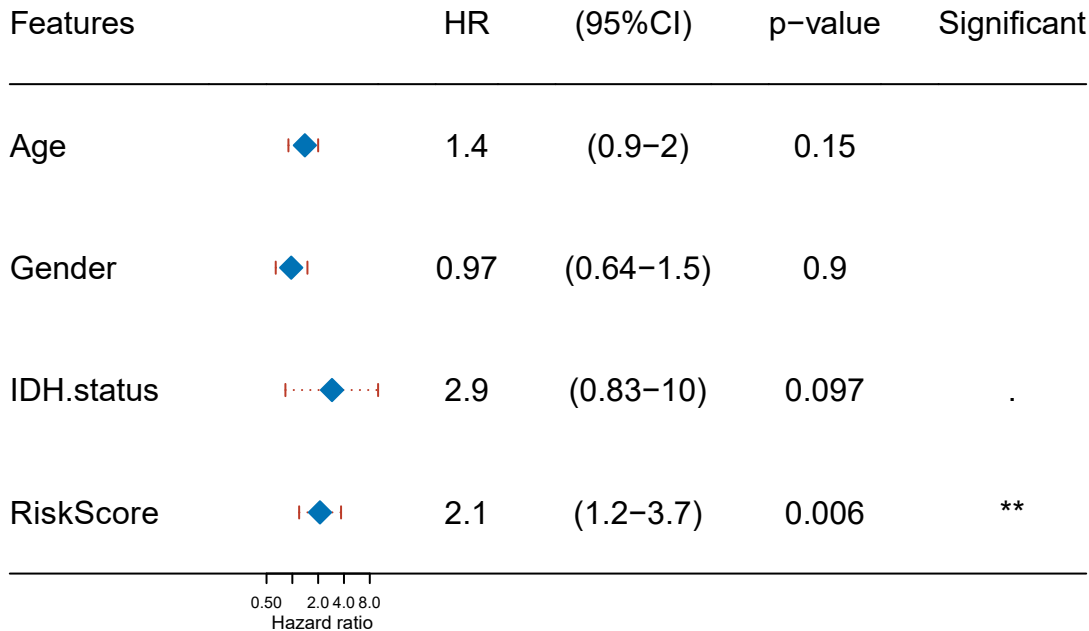

Supplement: Supplementary file 14 [file Image_6.PDF]

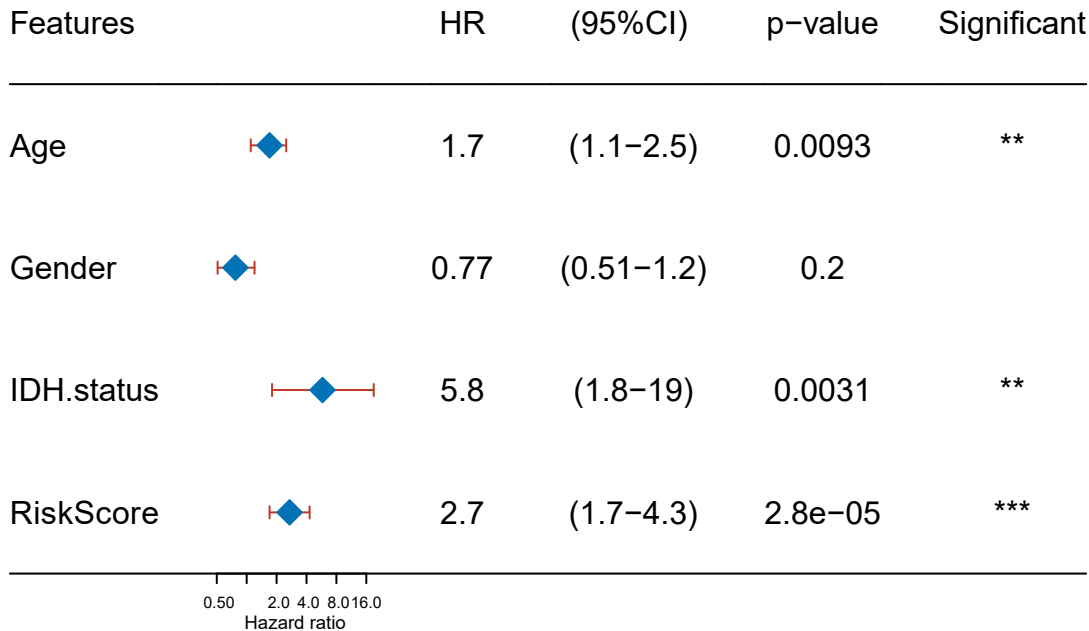

Supplement: Supplementary file 15 [file Image_7.PDF]

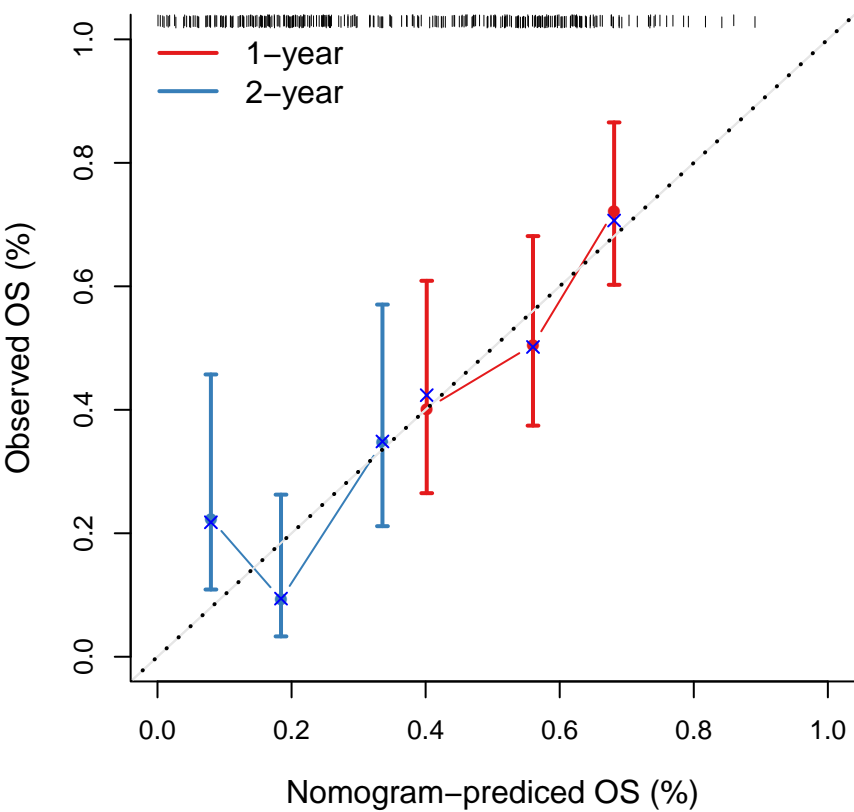

Points

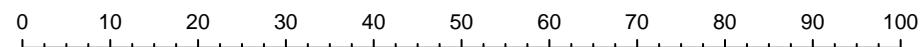

RiskScore

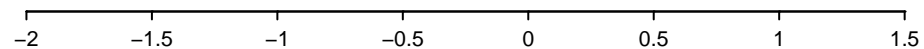

Total Points

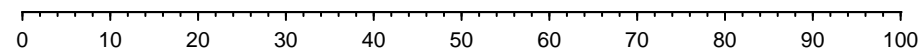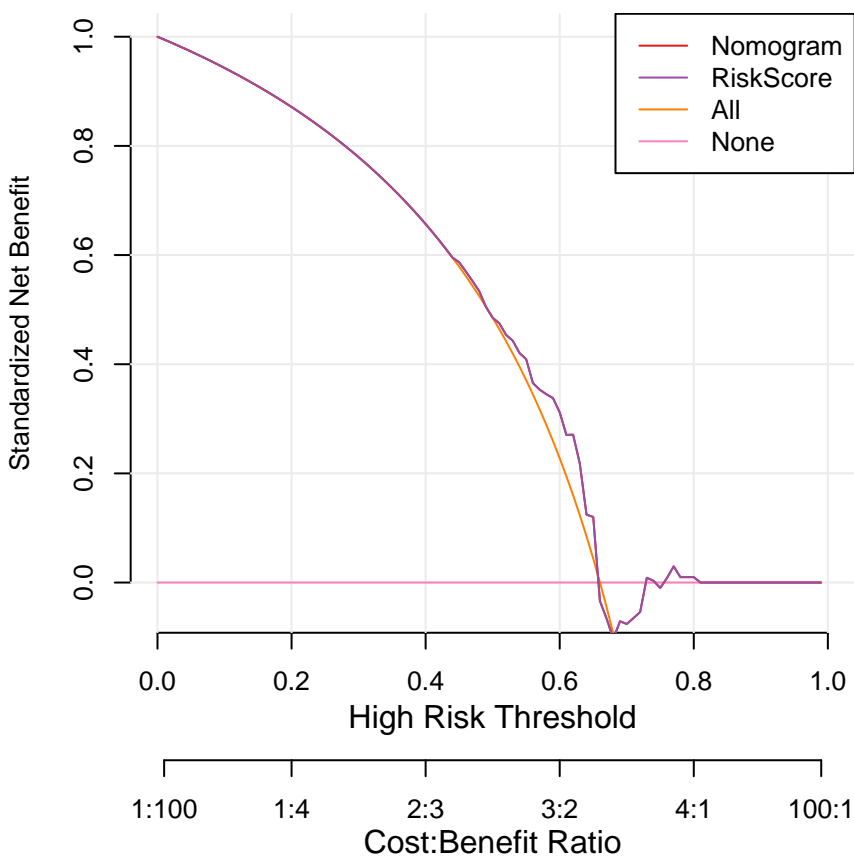

1-Year Survival

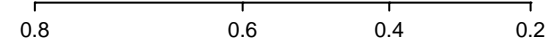

2-Year survival

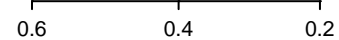

Supplement: Supplementary file 16 [file Image_8.PDF]
